# Candidate Resistant Genes of Sand Pear (*Pyrus pyrifolia* Nakai) to *Alternaria alternata* Revealed by Transcriptome Sequencing

**DOI:** 10.1371/journal.pone.0135046

**Published:** 2015-08-20

**Authors:** Xiaoping Yang, Hongju Hu, Dazhao Yu, Zhonghai Sun, Xiujuan He, Jingguo Zhang, Qiliang Chen, Rui Tian, Jing Fan

**Affiliations:** 1 School of Life Sciences, Wuhan University, Wuhan, Hubei, 430072, P. R. China; 2 Research Institute of Fruit and Tea, Hubei Academy of Agricultural Science, Wuhan, Hubei, 430064, P. R. China; 3 Hubei Laboratory of Crop Diseases, Insect Pests and Weeds Control, Wuhan, Hubei, 430064, P. R. China; Key Laboratory of Horticultural Plant Biology (MOE), CHINA

## Abstract

Pear black spot (PBS) disease, which is caused by *Alternaria alternata* (*Aa*), is one of the most serious diseases affecting sand pear (*Pyrus pyrifolia* Nakai) cultivation worldwide. To investigate the defense mechanisms of sand pear in response to *Aa*, the transcriptome of a sand pear germplasm with differential resistance to *Aa* was analyzed using Illumina paired-end sequencing. Four libraries derived from PBS-resistant and PBS-susceptible sand pear leaves were characterized through inoculation or mock-inoculation. In total, 20.5 Gbp of sequence data and 101,632,565 reads were generated, representing 44717 genes. Approximately 66% of the genes or sequenced reads could be aligned to the pear reference genome. A large number (5213) of differentially expressed genes related to PBS resistance were obtained; 34 microsatellites were detected in these genes, and 28 genes were found to be closely related to PBS resistance. Using a transcriptome analysis in response to PBS inoculation and comparison analysis to the PHI database, 4 genes (Pbr039001, Pbr001627, Pbr025080 and Pbr023112) were considered to be promising candidates for sand pear resistance to PBS. This study provides insight into changes in the transcriptome of sand pear in response to PBS infection, and the findings have improved our understanding of the resistance mechanism of sand pear to PBS and will facilitate future gene discovery and functional genome studies of sand pear.

## Introduction

Pear is one of the most important fruit crops in the world and has a long history of commercial cultivation in approximately 50 temperate countries [1–4]. However, pear diseases have become a major constraint on the pear industry worldwide [5]. Pear black spot (PBS) disease, which is caused by *Alternaria alternata* (*Aa*), is one of the most serious diseases in sand pear. PBS disease causes both necrosis on pear leaves, twigs, and fruits and early leaf drop, and it reduces productivity and the quality of fruit. PBS disease decreases pear yields in areas with high temperatures and humidity. The control of PBS disease is mainly achieved through the use of fungicides; however, large quantities of fungicides can produce potential toxic effects on humans and wildlife and lead to environment pollution [6, 7]. Moreover, the practice of applying large amounts of fungicides has increased the difficulty and cost of producing high-quality fruit [8]. Breeding disease-resistant varieties is the most effective and economical method of controlling the disease, although traditional breeding methods are hampered by the long growth period of pear [9]. However, the drawbacks of conventional breeding can be overcome by using plant resistance genes (R-gene) and molecular breeding approaches [10].

R-genes play an important role in plant genetic resistance mechanisms. A large number of R-genes have been isolated from several plant species, including rice, wheat, soybean, apple and grape [11–15]. Previous studies have attempted to isolate R-genes in pear, and four putative QTLs for fire-blight resistance were identified via the European pear genetic linkage map [16]. In addition, two genetic linkage maps of the pear scab resistance gene *Vnk* region of the cultivar Kinchaku have been constructed [17]. A leucine-rich repeat receptor-like protein kinase (LRPK) gene with possible involvement in scab resistance was identified from *Pyrus pyrifolia cv*. Kousui using reverse transcription-polymerase chain reaction (RT-PCR) and rapid amplification of cDNA ends PCR [18]. Approximately 100 disease-resistance gene candidates were identified from six species of pear based on conserved nucleotide-binding sites and leucine-rich repeat domains in resistance genes using a homology-based cloning method [5]. However, R-genes specific for PBS disease have yet to be cloned in pears. Thus, new approaches in pears, such as transcriptome sequencing, are required to identify an R-gene for PBS disease.

Transcriptome sequencing has proven to be an effective approach for analyzing changes in developmentally and environmentally induced gene expression at the transcriptome level [19]. Transcriptomic information has been used in a wide range of biological studies and provides fundamental insights into biological processes and applications such as gene expression levels [20], gene expression functional annotation during development or after experimental treatments [21, 22], gene discovery [23], SSR mining and SNP discovery [24]. This information can be used to help predict the roles and interactions of individual genes, discover more complex signal pathways activated in response to external stimuli and uncover potential cross-talk between these pathways. The latest paired-end tag sequencing strategy has further improved DNA sequencing efficiency and expands short-read lengths, thus providing a better depiction of the transcriptome [19].

In this study, differentially expressed genes (DEGs) of the pear in response to PBS infection were analyzed by paired-end transcriptome sequencing and available pear genome sequences (http://peargenome.njau.edu.cn:8004/default.asp?d=1&m=1) [25]. The objectives of this study were to screen R-genes that are differentially expressed in PBS-resistant and PBS-susceptible sand pear germplasms in response to *Aa* infection. The new dataset generated in this study will be a useful resource for future genetic and genomic studies of sand pear.

## Materials and Methods

### Plant Material and PBS Inoculation

Two sand pear germplasms that differ greatly in their resistance to *Aa* were used in this study. *Pyrus pyrifolia* Nakai. cv. ‘Jinjing’ pear (J), a cultivar with high resistance to *Aa* that was identified at the Wu Chang Sand Pear Garden National Fruit-tree Germplasm Resource (WCSPGNFGR) through three consecutive years (2009, 2010 and 2011) of field artificial inoculation experiments, was bred at the Research Institute of Fruit and Tea, Hubei Academy of Agricultural Science. *Pyrus pyrifolia* Nakai. cv. ‘Hongfen’ pear (H), a landrace germplasm resource with a high susceptibility to *Aa*, was also identified in the same field artificial inoculation experiments.

For the inoculations, inoculum production was conducted using a PBS strain (H) obtained from leaves of the Xiangnan pear from the germplasm bank of WCSPGNFGR. The H strain could infect the ‘Hongfen’ pear but not the ‘Jinjing’ pear. Stock cultures of the strain were stored at 4°C and sub-cultured in potato-sucrose-agar (PSA) medium at 28°C. After three weeks, 5 mL of sterilized distilled water was added to the surface of the colonies, which were scraped off using a small brush to remove the mycelium, and the conidial suspensions were filtered through four layers of cheesecloth. The conidial concentrate was centrifuged once for 20 min at 6000 g to remove mycelial fragments.

The conidial concentration was adjusted to 1 × 10^6^ spores mL^-1^. The spore suspension was sprayed onto detached young leaves of ‘Jinjing’ pear and ‘Hongfen’ pear with a glass atomizer. Control leaves of ‘Jinjing’ pear and ‘Hongfen’ pear were similarly sprayed with distilled water. The inoculated leaves were incubated in a moist chamber at 28°C. After inoculation with PBS, the leaves of ‘Jinjing’ pear and ‘Hongfen’ pear were sampled at 0 day, 1 day, 2 days, 3 days and 4 days. ‘Jinjing’ and ‘Hongfen’ pear leaves that were inoculated with distilled water were also sampled on these days.

### Sample Preparation, Read Alignment, and Sequence Analysis

Total RNA was extracted from each sample using TRIzol reagent (Invitrogen, Carlsbad, CA, USA) according to the manufacturer’s instructions. RNA purity was determined using a Nanodrop spectrophotometer (Thermo Fisher Scientific Inc., Wilmington, DE, USA), 1% formaldehyde gel electrophoresis, and a 2100 Bioanalyzer (Agilent Technologies, Santa Clara, CA, USA). The total RNA of each sample was diluted to 750 ng μl^-1^. The 200 μl mixture of RNA (‘Jinjing’ pear sample, J-P; and ‘Hongfen’ pear sample, H-P) used for sequencing included 40 μl of sample RNA of pear leaves inoculated with PBS at 0 day, 1 day, 2 days, 3 days and 4 days. Additionally, the 200 μl mixture of RNA (‘Jinjing’ pear sample, J-CK; and ‘Hongfen’ pear sample, H-CK) used for sequencing included 40 μl of sample RNA of pear leaves inoculated with distilled water at 0 day, 1 day, 2 days, 3 days and 4 days. The sample numbers H-CK, H-P, J-CK and J-P were used in the experimental design and data analysis.

The purity of the four RNA mixtures was determined using a Nanodrop spectrophotometer, 1% formaldehyde gel electrophoresis, and a 2100 Bioanalyzer. Oligo (dT)-coated beads were used to isolate poly (A) mRNA after the four RNA mixtures were collected. A fragmentation buffer (Ambion, Austin, TX, USA) was added to digest the mRNA to produce short fragments. First-strand cDNA synthesis was performed using random hexamer primers, and second-strand cDNA synthesis was performed using buffer, dNTPs, RNaseH and DNA polymerase I. Short fragments were purified with the QIAquick PCR Purification kit (Qiagen, Valencia, CA) for the end repair and poly (A) addition reaction. Subsequently, sequencing adapters were connected to the short fragments; the cDNA library was then constructed and purified. Finally, the sequencing library was constructed by PCR amplification and sequenced by Biomarker Company using an Illumina HiSeq 2000 sequencing platform.

Sequence reads were aligned using Bowtie (http://bowtie-bio.sourceforge.net), an ultrafast short-read mapping program, using the pear genome as a reference [25]. TopHat (http://tophat.cbcb.umd.edu) was used to identify the splice junctions, and raw digital gene expression data were normalized as reads per kilobase pair of transcript per million mapped reads (RPKM), with genes that had P-values <0.001 selected for further analysis. Expression data were log_2_ transformed and filtered at differences of 2-fold or greater in expression for each mixture sample. Differential patterns of gene expression from the various samples were represented by scatter diagrams.

### Sequence Annotation

The optimal assembly results were selected according to the assembly evaluation, and clustering analysis was performed to produce a DEG database composed of potential alternative splicing transcripts. The DEGs were annotated by alignment with those deposited in diverse protein databases, including the National Center for Biotechnology Information (NCBI) non-redundant protein (Nr) database, the NCBI non-redundant nucleotide sequence (Nt) database, the Swiss-Prot database, the Kyoto Encyclopedia of Genes and Genomes (KEGG) database, the Gene Ontology (GO) database, the Cluster of Orthologous Groups of proteins (COG) database and the TrEMBL database.

### SSR Mining and Primer Design

MIcroSAtellite (MISA) (http://pgrc.ipk-gatersleben.de/misa/) was employed for microsatellite mining. In this study, SSRs were considered to contain motifs with at least 10 contiguous repeats of one nucleotide, 6 repeats of two nucleotides, and 5 repeats of 3 to 6 nucleotides. Based on the MISA results, Primer3 software (http://primer3.sourceforge.net) was used to design the SSR primers, with the PCR product size ranging from 250 to 400 bp. In total, sixty-eight pairs of random SSR primers were used to analyze polymorphisms between PBS-resistant cultivar ‘Jinjing’ pear and PBS-susceptible ‘Hongfen’ pear cultivar.

### Validation of RNA-Seq Data by Quantitative Real-Time RT-PCR

Total RNA was reverse transcribed into first-strand cDNA using an M-MLV First Strand Kit (Invitrogen) according to the manufacturer’s instructions. Twenty-six genes were selected for confirmation by qRT-PCR with SYBR Premix Ex Taq (Takara, Japan). Primers for the chosen genes were designed using the Primer 6.0 program (PREMIER Biosoft International, Canada). The qRT-PCR gene expression analysis was performed on a StepOne real-time PCR System (Applied Biosystems, USA) using the glyceraldehyde-3-phosphate dehydrogenase (GAPDH) gene as an endogenous control [26]. Briefly, each reaction (final volume, 20 μl) contained 10 μl of 2 × TransStart Top Green qPCR SuperMix (TransGen, Beijing), 0.5 μl of passive reference dye II (50×), 0.4 μl (each) of forward and reverse primers (10 mM), 2 μl of cDNA template (corresponding to 50 ng total RNA), and 7 μl of RNase-free water. The reaction mixtures were heated to 94°C for 30 s, followed by 40 cycles at 94°C for 5 s, 55°C for 15 s, and 72°C for 34 s. A melting curve was generated for each sample at the end of each run to monitor the purity of the amplified products. Gene expression levels were analyzed using StepOne Software v2.0. The relative expression levels of target genes were calculated using the 2^−ΔΔCT^ method [27]. All of the samples were tested in triplicate, and the experiments were performed on three biological replicates.

## Results

### Symptoms in Leaves after PBS Inoculation

**Fig 1:**
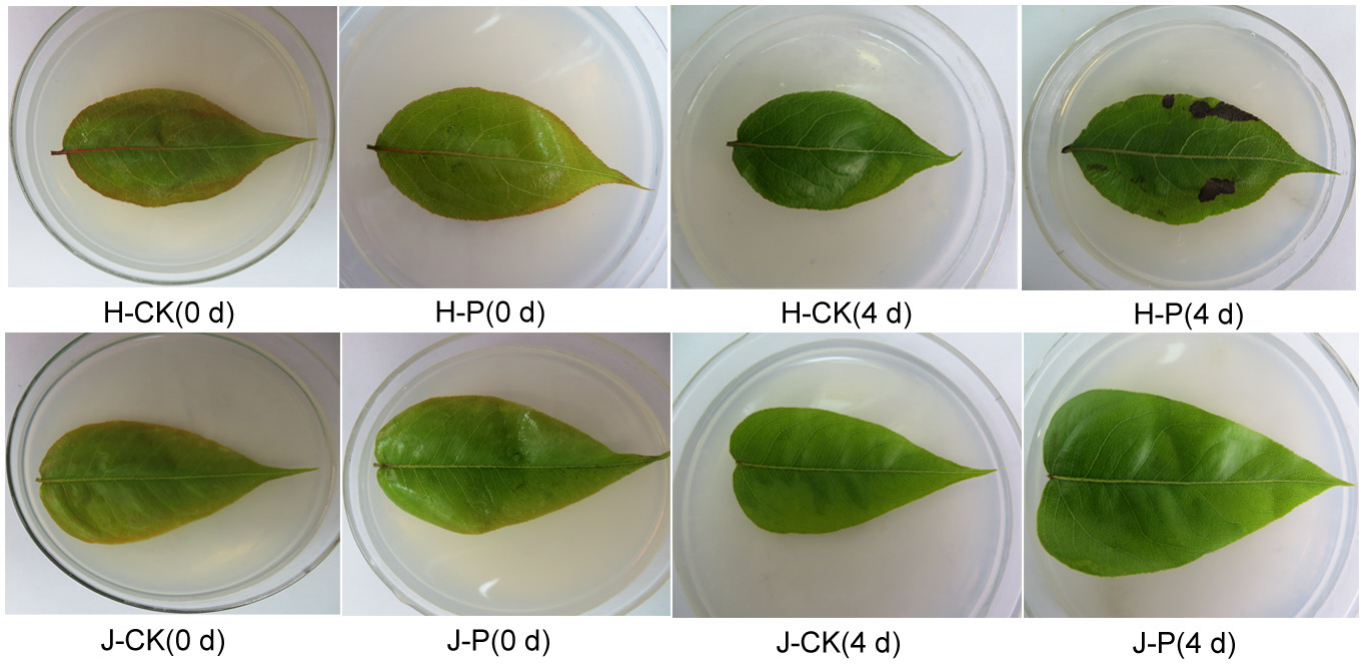
Disease symptoms in ‘Hongfen’ and ‘Jinjing’ pear after PBS inoculation. The leaves of ‘Hongfen’ pear inoculated with strain H presented a small brown necrosis at the early disease stage and large necrotic lesions on day 4, and the leaves of ‘Jinjing’ pear inoculated with strain H showed negligible necrotic spots.

The ‘Jinjing’ and ‘Hongfen’ pear cultivars showed different symptoms in response to PBS strain (H) after inoculation. ‘Jinjing’ pear leaves inoculated with strain H showed negligible necrosis on days 0, 1, 2, 3 and 4. However, ‘Hongfen’ pear leaves inoculated with strain H presented small brown necrotic spots on day 1. Leaf necrosis became more serious over time and developed into large necrotic lesions on day 4. ‘Jinjing’ and ‘Hongfen’ pear leaves inoculated with distilled water did not present necrotic spots on any day (Fig 1).

### Sequencing and Assembly of Illumina Short Reads

Four libraries (H-CK, H-P, J-CK and J-P) were analyzed by RNA sequencing (RNA-seq). H-CK and H-P RNA were derived from the leaves of ‘Hongfen’ pear inoculated with distilled water and the H strain, respectively. J-CK RNA and J-P RNA were derived from the leaves of ‘Jinjing’ pear inoculated with distilled water and the H strain, respectively. More than 25 million 100-bp paired-end reads, encompassing over 5 Gb of sequence data, were generated for each library (Table 1). The data sets supporting the results of this study are available at the NCBI SRA repository under accession number SRP051914.

**Table 1:** Evaluation statistics of sample sequencing data.

Sample	Total reads	Total nucleotides (bp)	CycleQ20 percentage	GC percentage
H-CK	25,033,955	5,056,458,588	100.00%	47.07%
H-P	25,410,160	5,132,229,853	100.00%	47.48%
J-CK	25,783,126	5,207,307,249	100.00%	47.55%
J-P	25,405,324	5,131,376,433	100.00%	47.74%

Approximately 66% of the sequenced reads (33 million reads) were successfully mapped to the pear genome reference sequence using Bowtie and TopHat software. Of these, 87.23% were uniquely mapped reads and 12.77% were multiple mapped reads in the H-CK sample (Table 2).

**Table 2:** Statistics of the reads and comparison to the pear reference genome.

Statistical content	H-CK		H-P		J-CK		J-P	
	Number	Percentage	Number	Percentage	Number	Percentage	Number	Percentage
Total reads	50,067,910	100%	50,820,320	100%	51,566,252	100%	50,810,648	100%
Mapped reads	33,129,244	66.17%	33,660,839	66.24%	34,355,267	66.62%	33,105,134	65.15%
Uniquely mapped reads	28,898,713	87.23%	29,569,456	87.85%	30,037,795	87.43%	28,880,457	87.24%
Multiple mapped reads	4,230,531	12.77%	4,091,383	12.15%	4,317,472	12.57%	4,224,677	12.76%
INDEL reads	5,821,656	17.57%	5,825,654	17.31%	6,139,899	17.87%	5,794,326	17.50%

### Transcriptome Analysis in Response to PBS Inoculation

The DEGs were analyzed using EBSeq software (Version 1.1.3) [28]. Using transcriptome sequencing, 5213 DEGs related to PBS resistance were obtained. A total of 909 DEGs were obtained between H-P and H-CK, and 501 DEGs were obtained between J-P and J-CK. A greater number of genes showed down-regulation following inoculation (Table 3), whereas a greater number of DEGs were screened between different varieties with either the water or PBS strain inoculation treatment. For example, 3460 DEGs were detected between H-CK and J-CK, whereas 3305 DEGs were obtained between H-P and J-P (Table 3). Scatter diagrams of gene expression are shown in S1 Fig.

**Table 3:** Statistics of the differentially expressed genes between the two samples.

Group	Number	Up number	Down number
H-CK vs J-CK	3,460	1,804	1,656
H-P vs J-P	3,305	1,735	1,570
H-P vs H-CK	909	364	545
J-P vs J-CK	501	209	292

The DEGs were grouped into distinct clusters based on expression patterns, and the results indicated that 3071 (88.8%) of the 3460 DEGs between H-CK and J-CK had significant matches in the Nr database, 3015 (87.1%) had significant matches in the Nt database, 2359 (68.2%) had significant matches in the Swiss-Prot database, 3071 (88.8%) had significant matches in the TrEMBL database, 2612 (75.5%) had significant matches in the GO database, 617 (17.8%) had significant matches in the KEGG database, and 1267 (36.6%) had significant matches in the COG database (Table 4).

**Table 4:** Statistics of the differentially expressed gene annotations.

Group	Nr	Nt	Swiss-Prot	TrEMBL	GO	KEGG	COG
H-CK_vs_J-CK	3,071	3,015	2,359	3,071	2,612	617	1,267
H-P_vs_H-CK	855	856	678	855	730	140	333
H-P_vs_J-P	2,925	2,897	2,229	2,923	2,466	567	1,162
J-P_vs_J-CK	469	453	372	469	399	75	160

The COG database classifies orthologous gene products. Every protein in COG is assumed to be evolved from an ancestor protein, and the entire database is built on coding proteins with complete genomes. The DEGs were aligned to the COG database to predict and classify possible functions. Among the 25 COG categories, the cluster for ‘General function prediction only' represented the largest group, followed by 'Transcription', 'Replication, recombination and repair' and 'Signal transduction mechanisms'. The cluster for 'Cell motility' represents the smallest group (S2, S3, S4 and S5 Figs).

Furthermore, GO functional annotation suggested that the DEGs can be categorized into 56 functional terms. A number of DEGs between the inoculation treatment and the water treatment in the same species were categorized into the 'cell killing' group. For example, 6 genes were categorized into the 'cell killing' group between H-P and H-CK, and 2 genes were categorized into the 'cell killing' group between J-CK and J-P (S6, S7, S8 and S9 Figs).

The KEGG PATHWAY database records networks of molecular interactions in these cells as well as their variants specific to particular organisms. Pathway-based analyses help to further elucidate the biological functions of genes. KEGG PATHWAY enrichment analysis showed that the most abundant categories in our analysis included 'Glycolysis/Gluconeogenesis,' 'Phenylalanine metabolism,' 'Plant hormone signal transduction,' 'Plant-pathogen interaction,' 'Ribosome,' and 'Spliceosome' (S10, S11, S12 and S13 Figs). These pathways might be significant in the resistance to *Aa* processes, especially in the 'Plant-pathogen interaction' metabolic pathway. Of the DEGs that participated in the 'Plant-pathogen interaction' metabolic pathway, 19 were between samples H-CK and J-CK, 23 were between samples H-P and J-P, 4 were between samples H-CK and H-P, and 1 was between samples J-CK and J-P.

**Fig 2:**
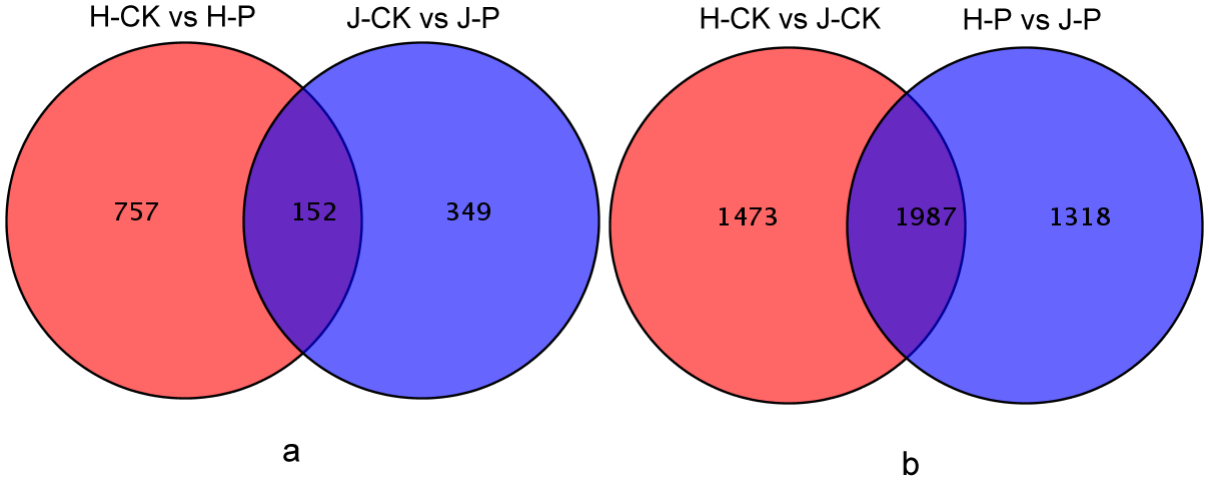
Number inside the parentheses indicates the number of DEGs [P<0.001 and fold change >2.0]. (a) Differentially expressed genes (DEGs) between ‘Hongfen’ pear (H-CK/H-P) inoculated with distilled water and PBS and ‘Jinjing’ pear (J-CK/J-P) inoculated with distilled water and PBS. (b) DEGs derived from different varieties receiving the same treatment; 1987 DEGs were detected in ‘Hongfen’ pear and ‘Jinjing’ pear from both the water treatment (H-CK/J-CK) and PBS inoculation (H-P/J-P).

We also compared the DEGs derived from ‘Hongfen’ (H-CK/H-P) with the DEGs from ‘Jinjing’ (J-CK/J-P), and 152 DEGs were observed for both varieties with PBS inoculation. These DEGs could represent the common genes induced by the PBS H strain. The DEGs derived from different varieties within the same treatment were compared, and 1987 DEGs were detected from the water treatment (H-CK/J-CK) and PBS inoculation (H-P/J-P) (Fig 2).

**Fig 3:**
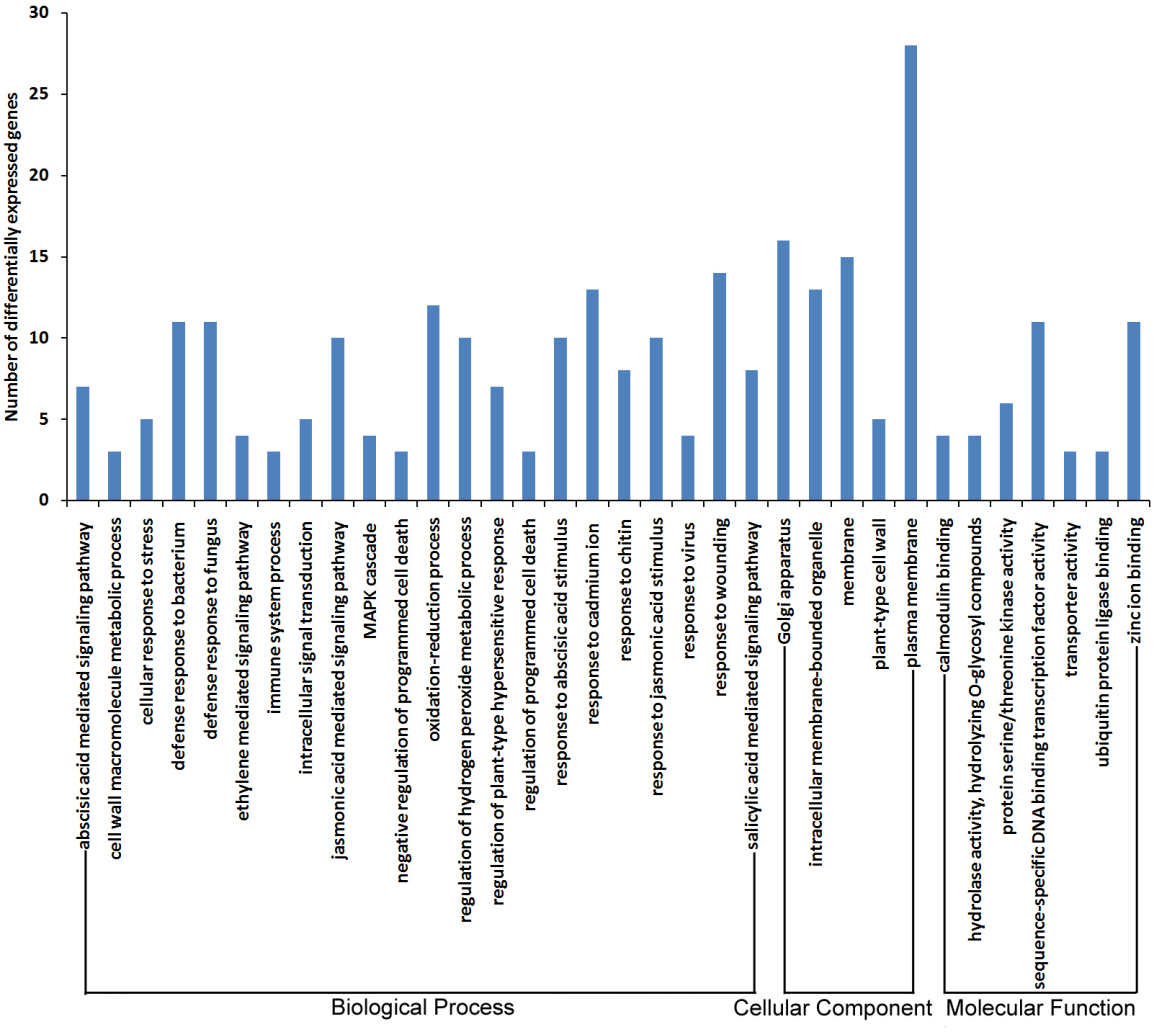
Functional annotation of 152 DEGs derived from ‘Jinjing’ (J-CK/J-P) compared with the DEGs from ‘Hongfen’ (H-CK/H-P) based on Gene Ontology (GO) analysis. GO analysis was performed for three main categories (cellular component, molecular function and biological process).

**Fig 4:**
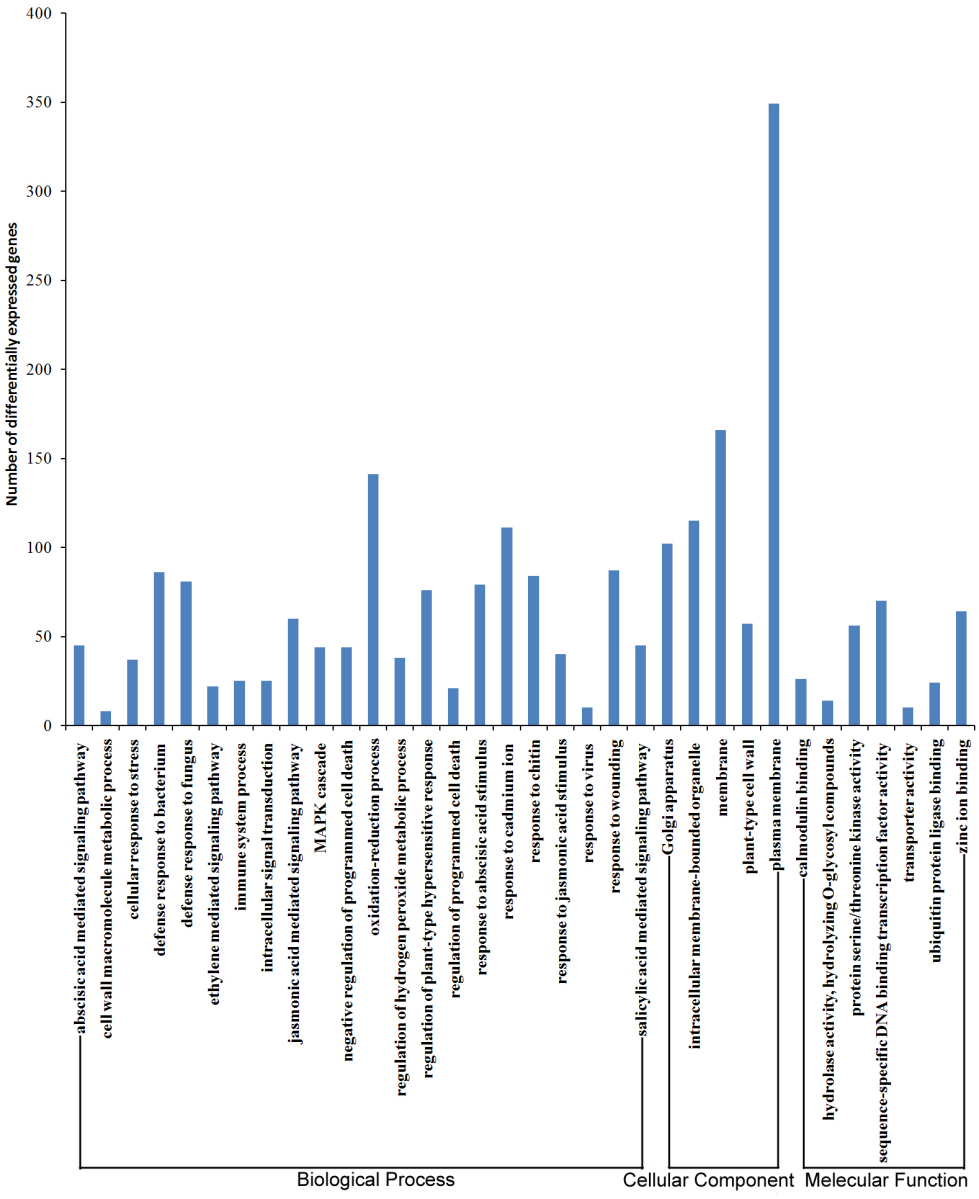
Functional annotation of 1987 DEGs derived from the water treatment (H-CK/J-CK) and PBS inoculation (H-P/J-P) based on Gene Ontology (GO) analysis. GO analysis was performed for three main categories (cellular component, molecular function and biological process).

Resistance induced by the pathogen and the resistance of sand pear cultivars were the focus of the data analysis. The 152 DEGs derived from ‘Jinjing’ (J-CK/J-P) compared with the DEGs from ‘Hongfen’ (H-CK/H-P) and the 1987 DEGs derived from the water treatment (H-CK/J-CK) and PBS inoculation (H-P/J-P) were categorized into 33 functional terms that were probably related to plant resistance to disease. For example, 11 genes were categorized into defense response to fungus (GO: 0050832), 13 were categorized into intracellular membrane-bounded organelle (GO: 0043231) and 4 were categorized into hydrolase activity, hydrolyzing O-glycosyl compounds (GO: 0004553) among the 152 DEGs (Fig 3). Furthermore, among the 1987 DEGs, 81 genes were categorized into defense response to fungus (GO: 0050832), 115 were categorized into intracellular membrane-bounded organelle (GO: 0043231) 14 genes were categorized into hydrolase activity, hydrolyzing O-glycosyl compounds (GO: 0004553) (Fig 4).

### SSR Mining from the Sand Pear Transcriptome

In this study, 34 microsatellites were detected in the 152 DEGs derived from ‘Jinjing’ (J-CK/J-P) compared with the DEGs from ‘Hongfen’ (H-CK/H-P), and 21 (61.8%) gene sequences contained 1 motif. The microsatellites included 9 (26.5%) dinucleotide motifs, 1 (2.9%) trinucleotide motif, and 3 (8.8%) compound motifs. A total of 68 primer pairs were successfully designed based on these 34 SSRs, 11 of which were able to distinguish between ‘Jinjing’ and ‘Hongfen’ pear (S1 Table). As the markers were developed from the 152 DEGs, which may represent genes related to resistance or susceptibility traits, these markers could be related to candidate genes involved in PBS resistance or susceptibility and could be useful for future efforts to breed resistance.

### Analysis of the Sand Pear Resistance Gene to *Aa*

From the transcriptome analysis in response to PBS inoculation, 5213 DEGs were obtained. From the functional annotation of 152 DEGs derived from ‘Jinjing’ (J-CK/J-P) compared with the DEGs from ‘Hongfen’ (H-CK/H-P) and the 1987 DEGs derived from the water treatment (H-CK/J-CK) and PBS inoculation (H-P/J-P), 28 genes with potential resistance to PBS were screened, including 16 nucleotide binding site-leucine-rich repeat (NBS-LRR) genes, 8 genes that encode resistance proteins and 4 genes related to transcription (S2 Table).

**Fig 5:**
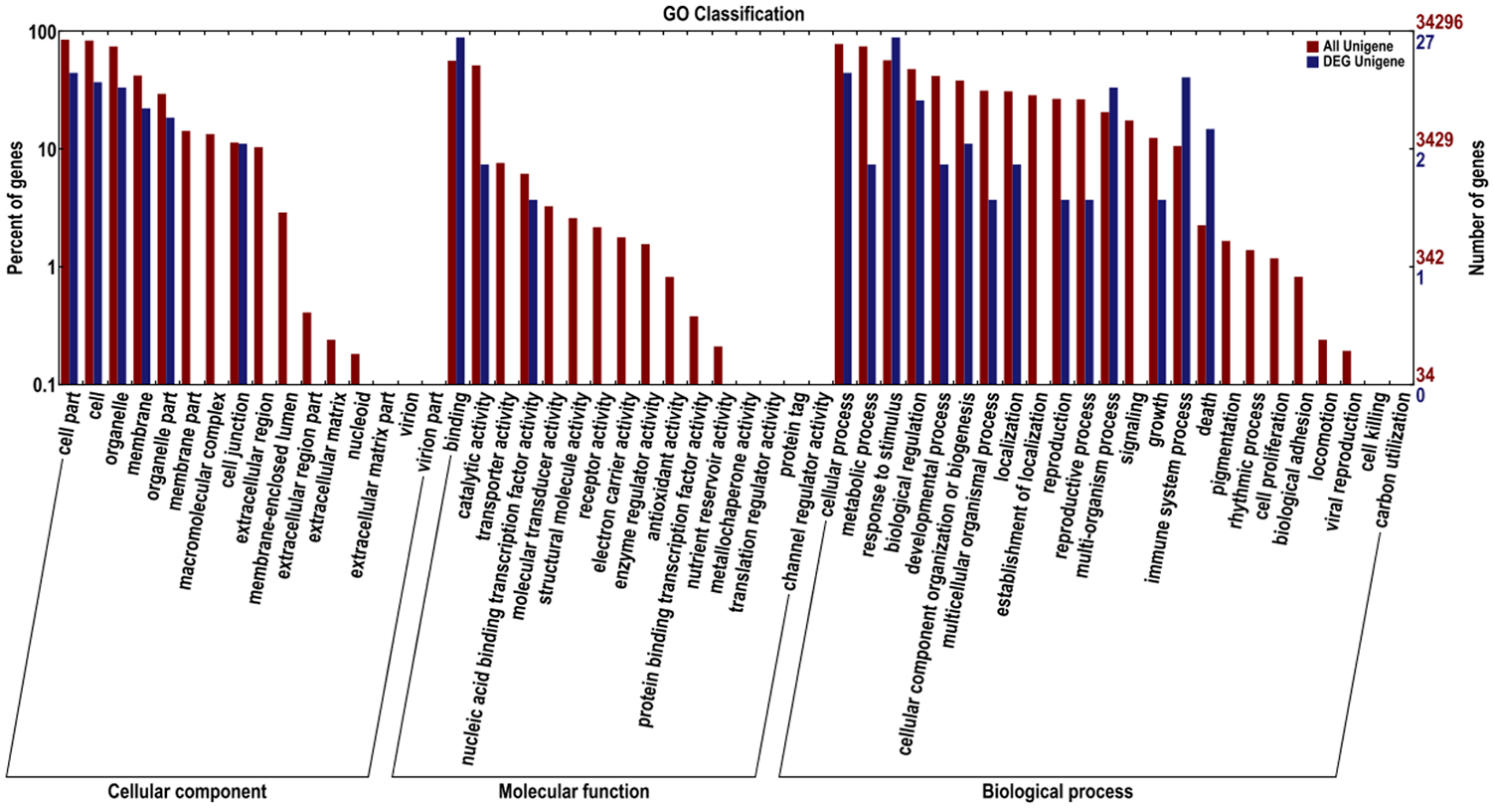
Functional annotation of 28 genes related to pear black spot resistance based on Gene Ontology (GO) analysis. GO analysis was performed for three main categories (cellular component, molecular function and biological process). To the left of the ordinate is the percentage of gene quantities, and to the right of the ordinate is the number of genes. Functional annotation of all assembled genes is indicated using a red color column, and the functional annotation of the differentially expressed genes is indicated using the blue color column.

In addition, the 28 DEGs were annotated based on the GO and COG databases. The GO categories of the 28 genes showed that 'differences in response to stimulus', 'signal transduction' and 'immune process and death process' were the top 4 significantly enriched categories (Fig 5). The classification was distinguished by orthologous gene products, and the functionality of each category was annotated using the COG database. Seven genes (Pbr023112, Pbr023278, Pbr025080, Pbr033741, Pbr039001, Pbr040608 and Pbr008283) were annotated with COG functions. Pbr025080 was associated with transcription, and the protein product of the other genes belonged to the unknown functional annotation in the COG database (S14 Fig).

**Fig 6:**
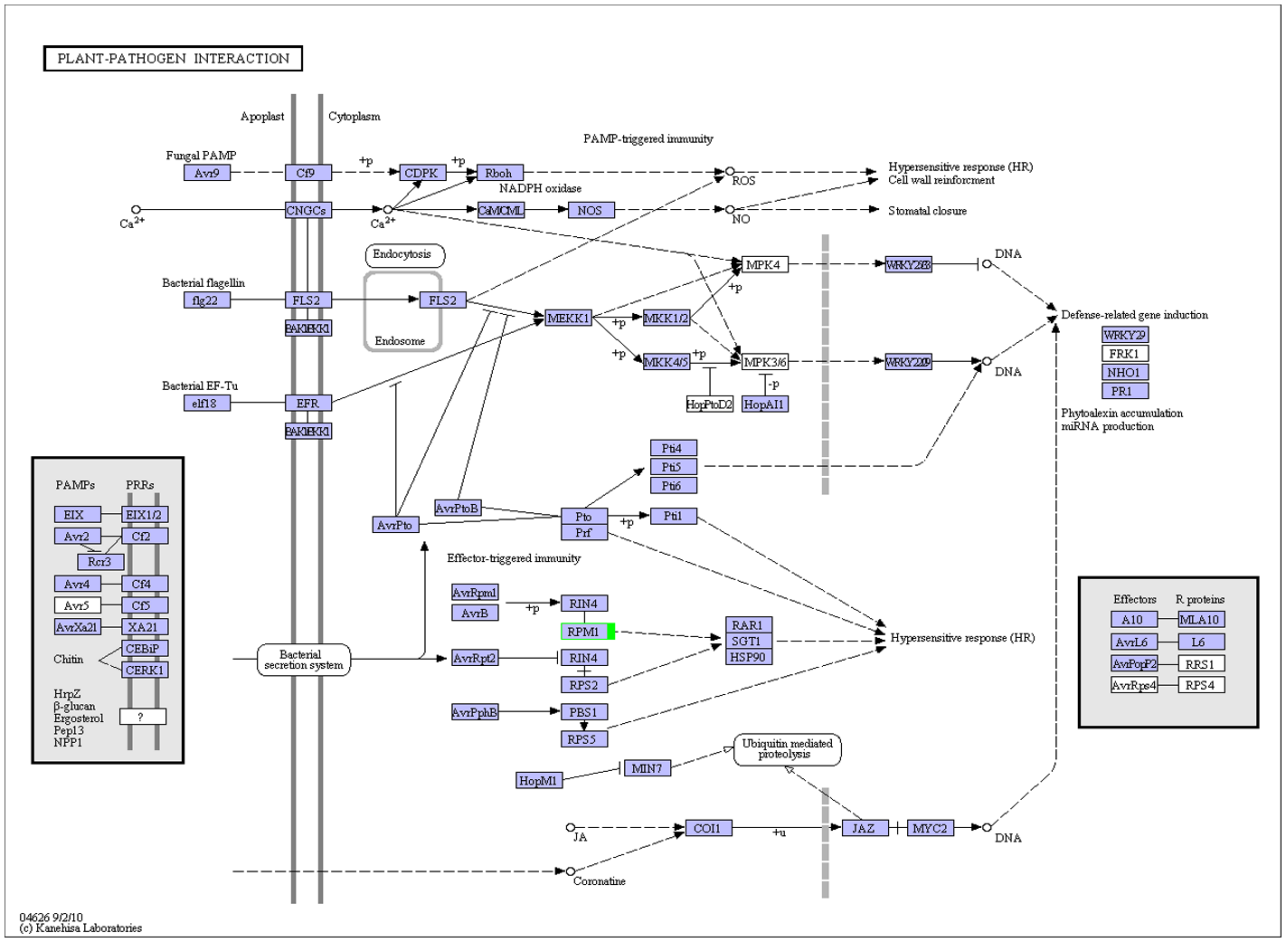
KEGG metabolic pathway of Pbr039001 related to pear black spot resistance. Frame numbers (instead of the enzyme number) indicate the corresponding genes associated with this enzyme. The pathway includes a number of different enzymes formed by complex biochemical reactions. Genes associated with this pathway are marked with different colored boxes: the red box is up-regulated genes, the green box is down-regulated genes, and the blue box is both up-regulated and down-regulated genes.

To further demonstrate the relationship between the 28 DEGs and PBS resistance, we identified metabolic pathways represented by the DEG collection. Annotations of 28 DEGs were fed into the KEGG Pathway Tools, which is an alternative approach to categorizing genes functions that emphasizes metabolic pathways. This process predicted the metabolic pathways represented by the 28 DEGs. An enrichment analysis of the 28 genes showed that the Pbr039001 gene encoded the disease resistance protein RPM1, which was significantly enriched in the plant pathogen interaction pathway (P-value = 0.0328) (Fig 6).

The Pathogen Host Interaction database (PHI-base) contains molecular and biological information on genes shown to affect the outcome of pathogen-host interactions (http://www.phi-base.org/). Twenty-two genes were successfully aligned to PHI-base R-gene sequences in the 28 genes related to PBS resistance, and the scores of 4 genes (Pbr001627 is 211, Pbr025080 is 193, Pbr023112 is 112, and Pbr025376 is 102) were higher than 100 (Table 5).

**Table 5:** Results for the sand pear genes resistant to *Aa* compared with data from the PHI-base.

Gene ID	Annotation	E_value	Identity	Score
Pbr001627	PHI:2389	6.21E-57	106/281(37.72)	211
Pbr025080	PHI:2389	2.34E-51	103/282(36.52)	193
Pbr023112	PHI:2389	2.99E-26	148/583(25.39)	112
Pbr025376	PHI:2389	5.03E-23	156/666(23.42)	102
Pbr023278	PHI:2390	1.04E-18	127/554(22.92)	88
Pbr000681	PHI:2390	3.13E-17	148/678(21.83)	84
Pbr001247	PHI:2389	3.71E-15	94/341(27.57)	78
Pbr022874	PHI:2391	3.08E-15	80/318(25.16)	78
Pbr041724	PHI:2389	1.88E-15	101/417(24.22)	78
Pbr000678	PHI:2389	1.12E-14	93/366(25.41)	76
Pbr012560	PHI:2389	7.55E-15	77/279(27.60)	76
Pbr022876	PHI:2389	5.14E-14	69/239(28.87)	75
Pbr038352	PHI:2391	1.03E-14	125/562(22.24)	75
Pbr023136	PHI:2390	2.81E-13	125/586(21.33)	71
Pbr033741	PHI:2389	2.76E-11	49/152(32.24)	64
pear_newGene_2229	PHI:2389	6.38E-11	37/106(34.91)	64
Pbr008283	PHI:2389	8.79E-11	65/218(29.82)	63
pear_newGene_1053	PHI:2390	1.71E-12	30/87(34.48)	58
Pbr035730	PHI:2390	6.25E-08	71/314(22.61)	56
Pbr007974	PHI:2391	4.13E-08	28/89(31.46)	53
Pbr039001	PHI:81	4.90E-07	49/176(27.84)	51
Pbr012606	PHI:2390	2.17E-06	41/125(32.80)	47

### RNA-Seq Expression Validation by qPCR

**Fig 7:**
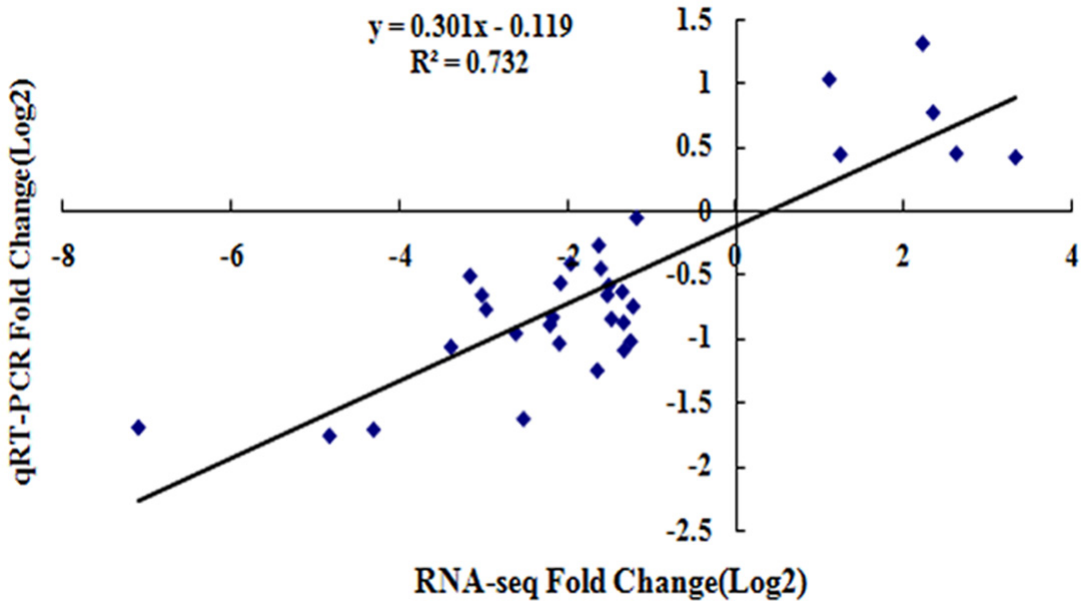
Real-time quantitative RT-PCR confirms 26 differentially expressed genes. The scatter plot shows expression changes (log2 fold) measured by RNA-seq and qRT-PCR analyses of the selected genes. Results are plotted for genes that showed significant (P≤0.05) up- or down-regulation. A linear trend line is shown.

To quantitatively determine the reliability of our transcriptome data, we monitored the expression of 26 DEGs by qRT-PCR. These genes included 22 DEGs related to PBS resistance and 4 regulatory proteins (Pbr020071 represents a RING finger protein, Pbr037418 represents a jasmonate-zim-domain protein, Pbr040066 represents a small heat shock protein, and Pbr042781 represents an NADP-dependent D-sorbitol-6-phosphate dehydrogenase) (S3 Table). The results of the qRT-PCR analysis revealed expression patterns that were consistent with the Illumina sequencing, although quantitative differences in expression levels were observed. The correlation between the RNA-seq and qRT-PCR results was measured by constructing a scatter plot of the log2 fold changes, and a positive correlation coefficient was observed (Pearson coefficient R^2^ = 0.732) (Fig 7).

## Discussion

The pear (*P*. *bretschneideri Rehd*. cv. ‘Dangshansuli’) genome, which encodes 42812 protein-coding genes, was released in 2013 [25]. In the present study, of the 44717 genes identified by transcriptome sequences, 41497 (92.8%) were successfully aligned to the pear genome reference sequence. Moreover, 3220 genes were identified as new pear genes, and 2206 of the new genes were annotated in the database. Among these, 1661 genes were matched in the Nr database, 870 were matched in the Swiss-Prot database, 1918 were matched in the Nt database, 1093 were matched in the GO database, 1661 were matched in the TrEMBL database, 243 were matched in the KEGG database, and 241 were matched in the COG database.

A total of 396 nucleotide-binding site (NBS)-containing R genes were identified in the pear genome [25], and 4 new NBS-containing R genes (pear_newGene_2235, pear_newGene_1053, pear_newGene_1262 and pear_newGene_2229) were identified by transcriptome sequencing in the present study.

Pathogens proliferate within plant tissues through the secretion of effector virulence-associated proteins, RNAs and metabolites that down-regulate basal defenses. In turn, plants employ myriad defense mechanisms to prevent pathogen proliferation [29]. Plants have evolved sophisticated surveillance systems to recognize pathogen effectors delivered into host cells. In this study, a KEGG enrichment analysis showed that the Pbr039001 gene encoded disease resistance protein RPM1. RPM1 participates in plant immune responses, is an NBS-LRR immune receptor that recognizes the *Pseudomonas syringae* effectors AvrB and AvrRpm1, and may guard against pathogens by using AvrRpm1 and AvrB to manipulate RIN4 activity [30, 31]. Pathogens inject pathogenic proteins directly into plant cells through the secretion system of the plant cell wall. However, plants are capable of activating a hypersensitivity response through the RPM1 protein stress response, and programmed cell death in the plant is employed to inhibit pathogen growth.

**Fig 8:**
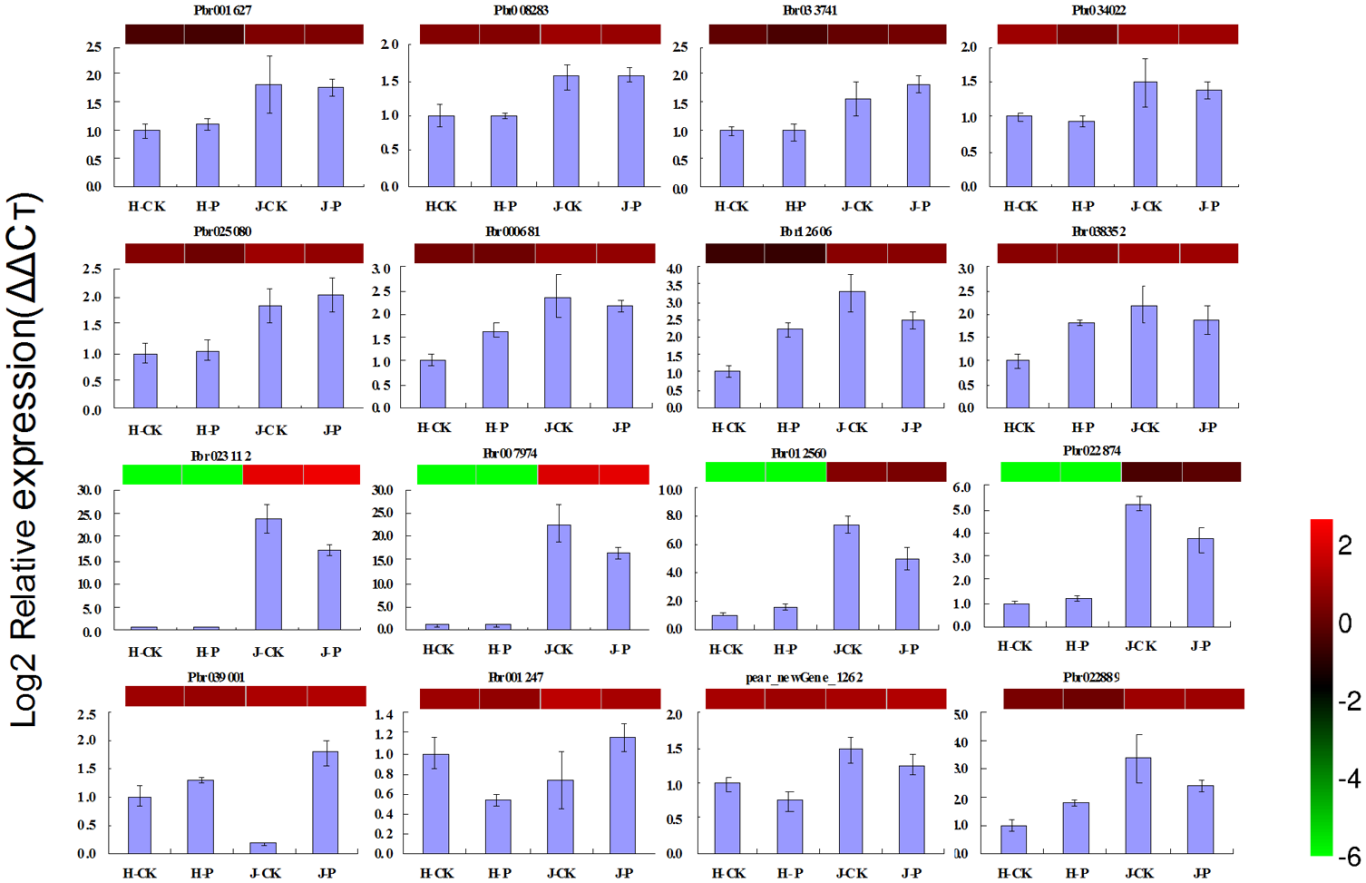
Expression profiles of 16 genes related to pear black spot resistance as determined by RNA-seq and qRT-PCR. Color panels above the bars represent log2-transformed mean expression values obtained by RNA-seq (see reference color bar).

Based on the RNA-seq and qRT-PCR analyses, 16 genes were divided into 4 groups (Fig 8). The first group contained 4 genes (Pbr023112, Pbr007974, Pbr012560 and Pbr022874) that were highly expressed in the resistant variety (‘Jinjing’ pear) but were minimally expressed in the susceptible variety (‘Hongfen’ pear). The fold difference in expression was more than 4, especially for Pbr023112, where the fold difference in expression was more than 20. The second group contained 4 genes (Pbr025080, Pbr000681, Pbr12606 and Pbr038352) that were more highly expressed in the resistant variety (‘Jinjing’ pear) than in the susceptible variety (‘Hongfen’ pear), and the fold difference in expression was between 2 and 3. The third group contained 4 genes (Pbr001627, Pbr008283, Pbr033741 and Pbr034022) that were more highly expressed in the resistant variety (‘Jinjing’ pear) than in the susceptible variety (‘Hongfen’ pear), and the fold difference in expression was less than 2. The fourth group contained 4 genes (Pbr039001, Pbr001247, pear_newGene_1262 and Pbr022889) that were differentially expressed in response to PBS inoculation, especially Pbr039001, whose fold difference in expression was more than 10.

Based on the GO functional annotation, COG function classification, KEGG analysis, and R gene comparison in the PHI-base of the 28 DEGs, 4 genes (Pbr039001, Pbr001627, Pbr025080 and Pbr023112) were screened as candidates with resistance to PBS. Pbr039001 mainly participated in biological plant-type hypersensitive response processes (GO: 0009626); Pbr001627 mainly participated in biological regulation of cellular processes (GO: 0050794), defense responses to bacterium (GO: 0042742), primary metabolic processes (GO: 0044238), innate immune responses (GO: 0045087), aromatic compound biosynthetic processes (GO: 0019438), and small molecule biosynthetic processes (GO: 0044283); Pbr025080 mainly participated in biological defense responses (GO: 0006952), cellular processes (GO: 0009987), and multi-organism processes (GO: 0051704); and Pbr023112 mainly participated in biological defense responses (GO: 0006952). The methods by which these 4 candidate genes function in the sand pear can be verified through transgenic experiments in future research.

## Conclusions

In conclusion, this work presents the first transcriptome sequencing analysis of resistant sand pear cultivars and susceptible germplasm leaves inoculated with PBS using the Illumina platform. In total, 20.5 Gbp of sequence data and 101,632,565 reads were generated, and approximately 66% of the sequenced reads were successfully aligned to the pear genome reference sequence. In summary, 44717 genes were identified by transcriptome sequences in this study, 5213 DEGs related to PBS resistance were obtained, 34 microsatellites were detected in the DEGs, and 107525 reliable SNPs were identified through transcriptome sequencing. From the transcriptome analysis of PBS inoculation, 28 DEGs related to PBS resistance were identified, and 4 genes (Pbr039001, Pbr001627, Pbr025080 and Pbr023112) were screened as candidates for sand pear resistance to *Aa*. These data will facilitate gene discovery and functional genomic studies in sand pear, and the findings will improve our understanding of the resistance mechanisms of sand pear to PBS. Therefore, this study provides insight into the complex transcriptome of the sand pear and establishes a biotechnological platform for future research.

## Supporting Information

S1 Fig Scatter diagrams of gene expression. (a) Scatter diagram of genes for H-CK and H-P using RPKM logarithm values. (b) Scatter diagram of genes for H-CK and J-CK using RPKM logarithm values. (c) Scatter diagram of genes for J-CK and J-P using RPKM logarithm value. (d) Scatter diagram of genes for H-P and J-P using RPKM logarithm values. The average RPKM logarithm values of genes in the two samples are indicated on the abscissa. The logarithm values of the differentially expressed genes in the two samples are indicated on the ordinate, which highlight the differentially expressed genes. Differentially expressed genes are indicated using blue dots, non-significantly differentially expressed genes are indicated using red dots.
(TIF)

S2 Fig Clusters of the expressed genes between samples H-CK and H-P based on COG classification. (TIF)

S3 Fig Clusters of the expressed genes between samples J-CK and J-P based on COG classification. (TIF)

S4 Fig Clusters of the expressed genes between samples H-CK and J-CK based on COG classification. (TIF)

S5 Fig Clusters of the expressed genes between samples H-P and J-P based on COG classification. (TIF)

S6 Fig Functional annotation of assembled sequences based on the Gene Ontology (GO) categorization for the expressed genes between samples H-CK and H-P. (TIF)

S7 Fig Functional annotation of assembled sequences based on the Gene Ontology (GO) categorization for the expressed genes between samples J-CK and J-P. (TIF)

S8 Fig Functional annotation of assembled sequences based on the Gene Ontology (GO) categorization for the expressed genes between samples H-CK and J-CK. (TIF)

S9 Fig Functional annotation of assembled sequences based on the Gene Ontology (GO) categorization for the expressed genes between samples H-P and J-P. (TIF)

S10 Fig KEGG classification chart of the differentially expressed genes between samples H-CK and H-P. (TIF)

S11 Fig KEGG classification chart of the differentially expressed genes between samples H-CK and J-CK. (TIF)

S12 Fig KEGG classification chart of the differentially expressed genes between samples J-CK and J-P. (TIF)

S13 Fig KEGG classification chart of the differentially expressed genes between samples H-P and J-P. (TIF)

S14 Fig Clusters of 28 differentially expressed genes (COG) classification. (TIF)

S1 Table 11 primer pairs which were able to distinguish between ‘Jinjing’ and ‘Hongfen’ pear were successfully designed based on 34 SSRs. (DOCX)

S2 Table Functional annotation of 28 differentially expressed genes related to PBS resistance. (DOCX)

S3 Table Primers used for real-time quantitative RT-PCR for the verification of Illumina data. (DOCX)
