# Supplementary figures and images for: Candidate Resistant Genes of Sand Pear (Pyrus pyrifolia Nakai) to Alternaria alternata Revealed by Transcriptome Sequencing
Source: PLoS One. 2015 Aug 20;10(8):e0135046. doi: 10.1371/journal.pone.0135046 (PMC4546377; doi:10.1371/journal.pone.0135046)

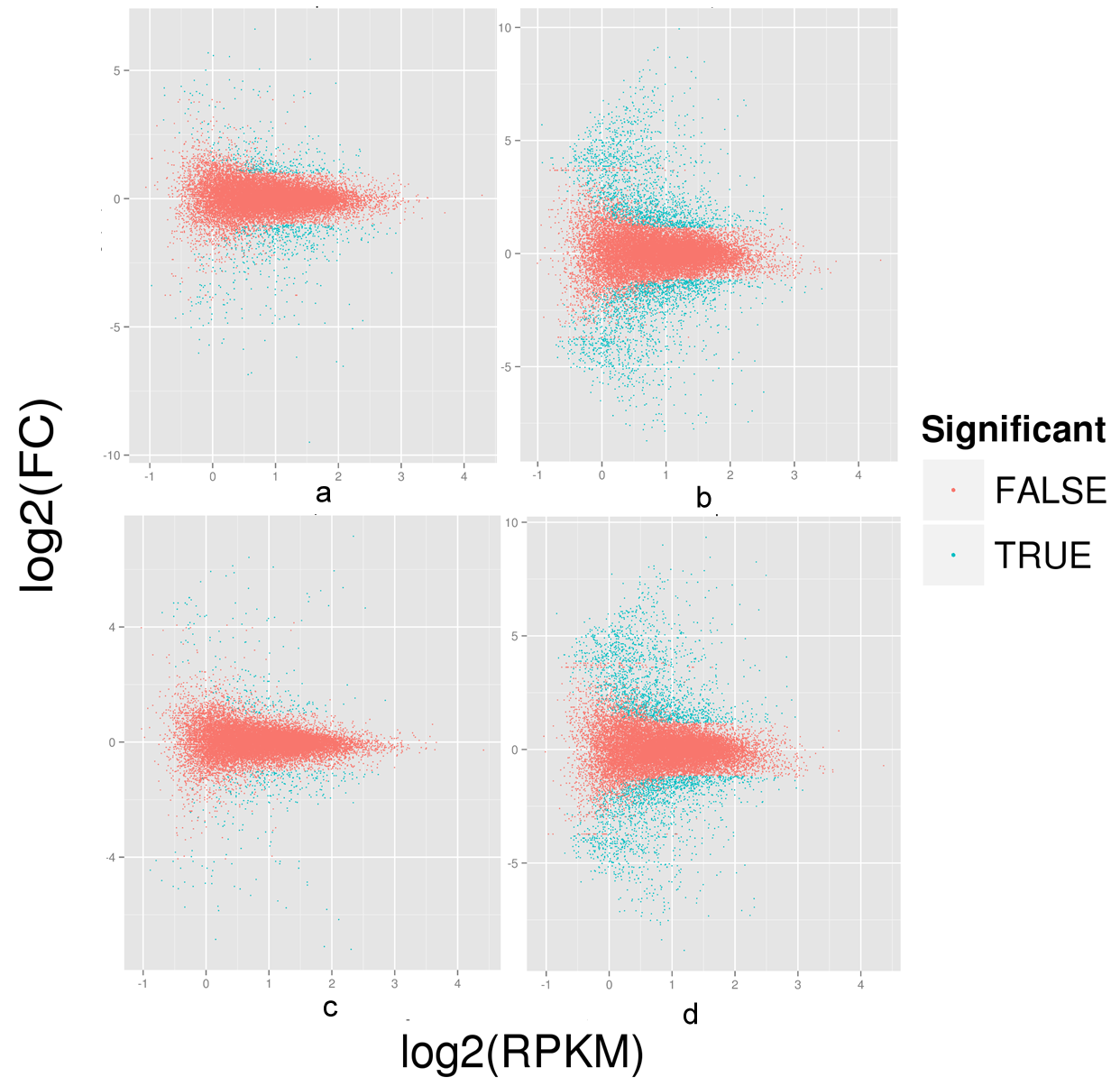

Supplement: S1 Fig — (a) Scatter diagram of genes for H-CK and H-P using RPKM logarithm values. (b) Scatter diagram of genes for H-CK and J-CK using RPKM logarithm values. (c) Scatter diagram of genes for J-CK and J-P using RPKM logarithm value. (d) Scatter diagram of genes for H-P and J-P using RPKM logarithm values. The average RPKM logarithm values of genes in the two samples are indicated on the abscissa. The logarithm values of the differentially expressed genes in the two samples are indicated on the ordinate, which highlight the differentially expressed genes. Differentially expressed genes are indicated using blue dots, non-significantly differentially expressed genes are indicated using red dots. (TIF) [file pone.0135046.s001.tif]

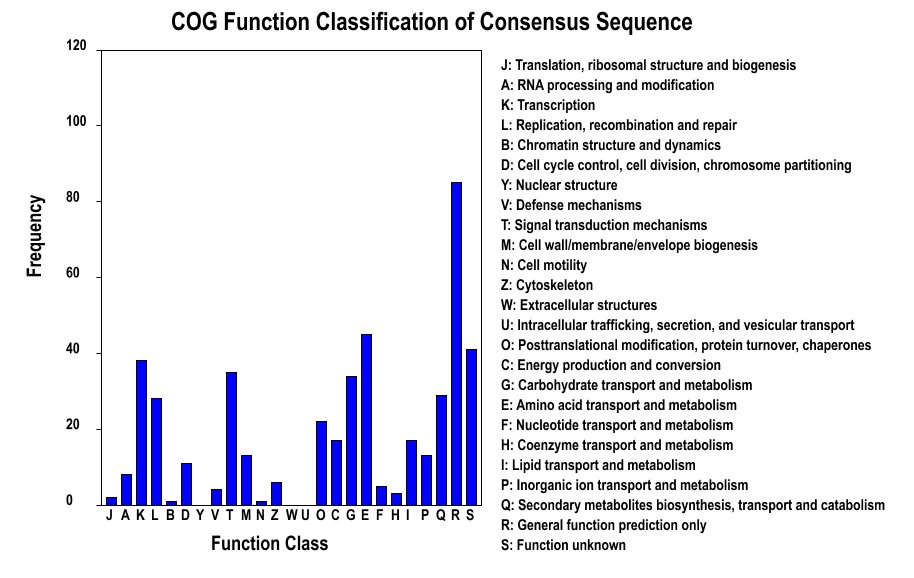

Supplement: S2 Fig — (TIF) [file pone.0135046.s002.tif]

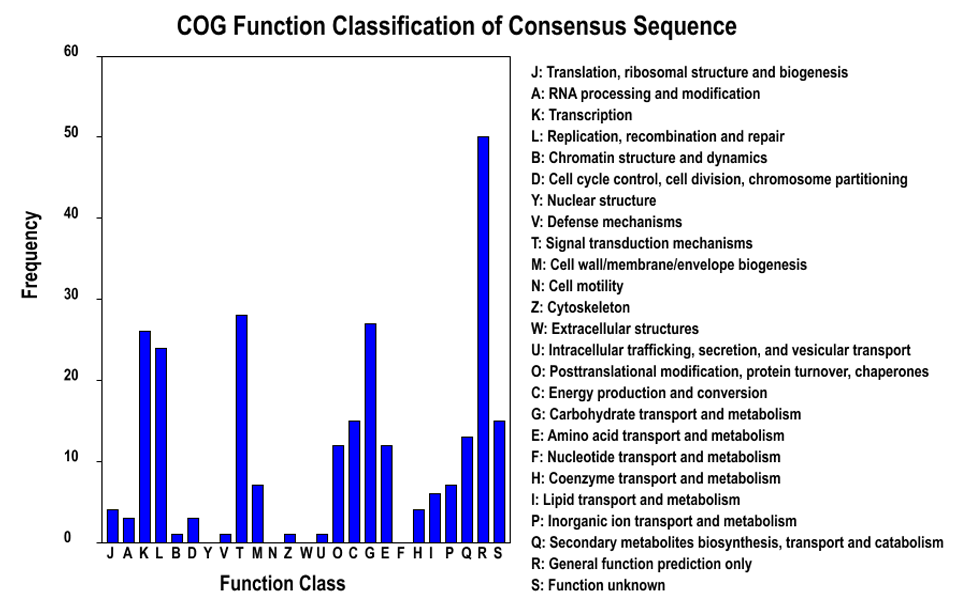

Supplement: S3 Fig — (TIF) [file pone.0135046.s003.tif]

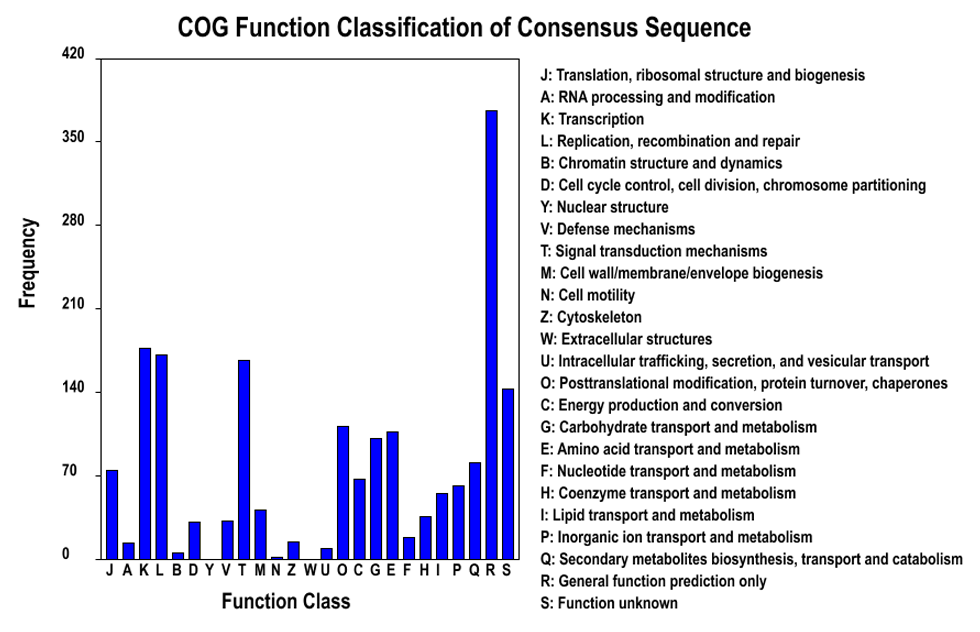

Supplement: S4 Fig — (TIF) [file pone.0135046.s004.tif]

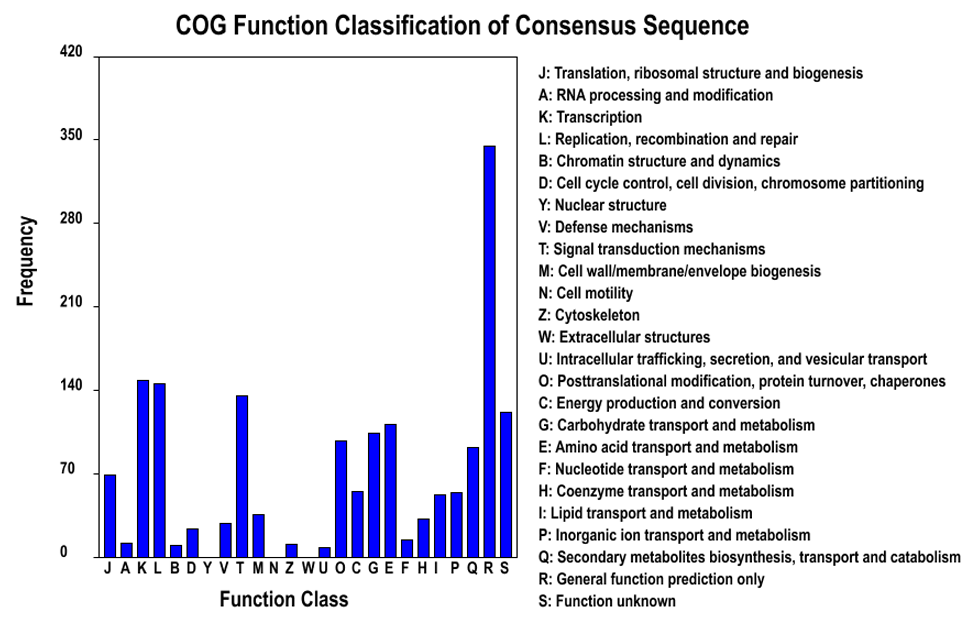

Supplement: S5 Fig — (TIF) [file pone.0135046.s005.tif]

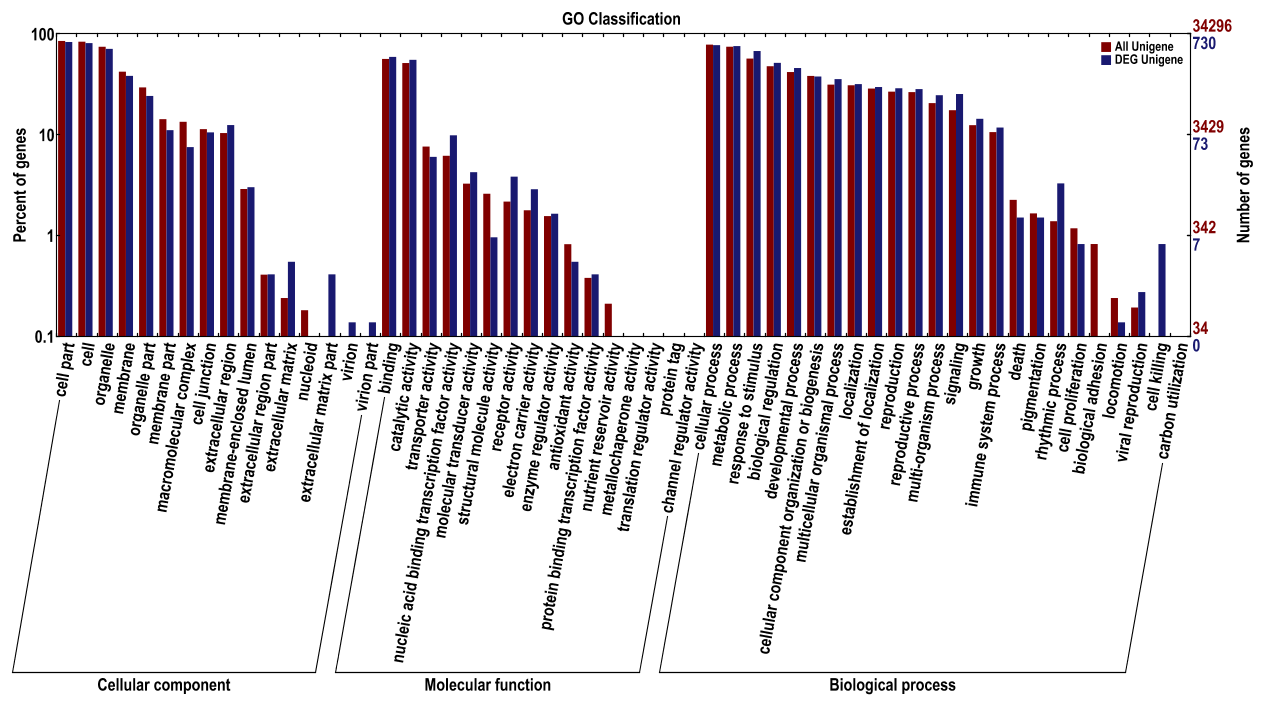

Supplement: S6 Fig — (TIF) [file pone.0135046.s006.tif]

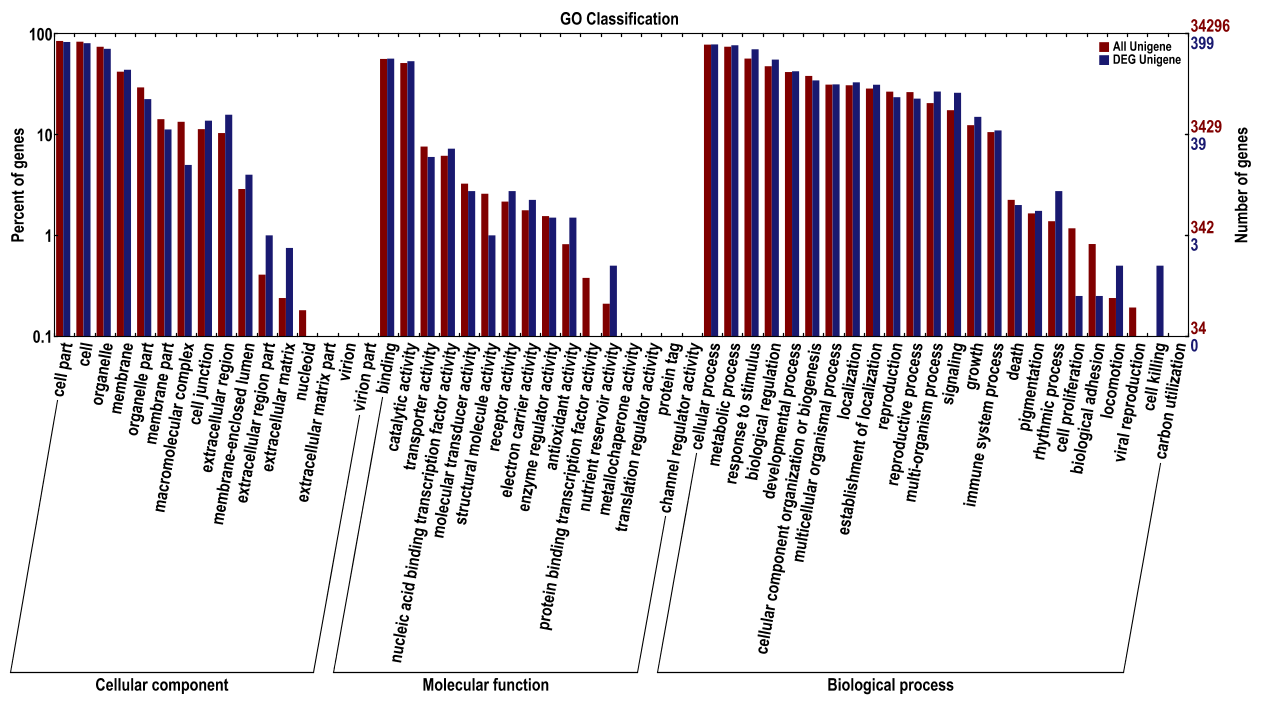

Supplement: S7 Fig — (TIF) [file pone.0135046.s007.tif]

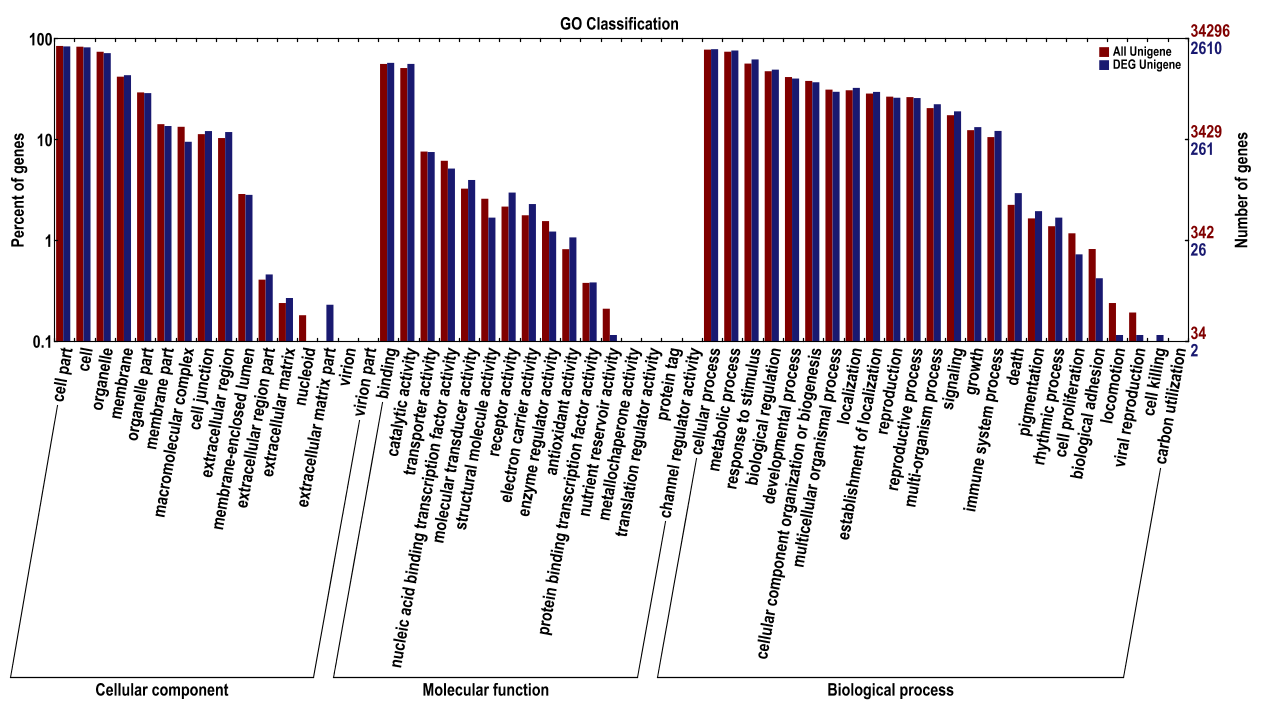

Supplement: S8 Fig — (TIF) [file pone.0135046.s008.tif]

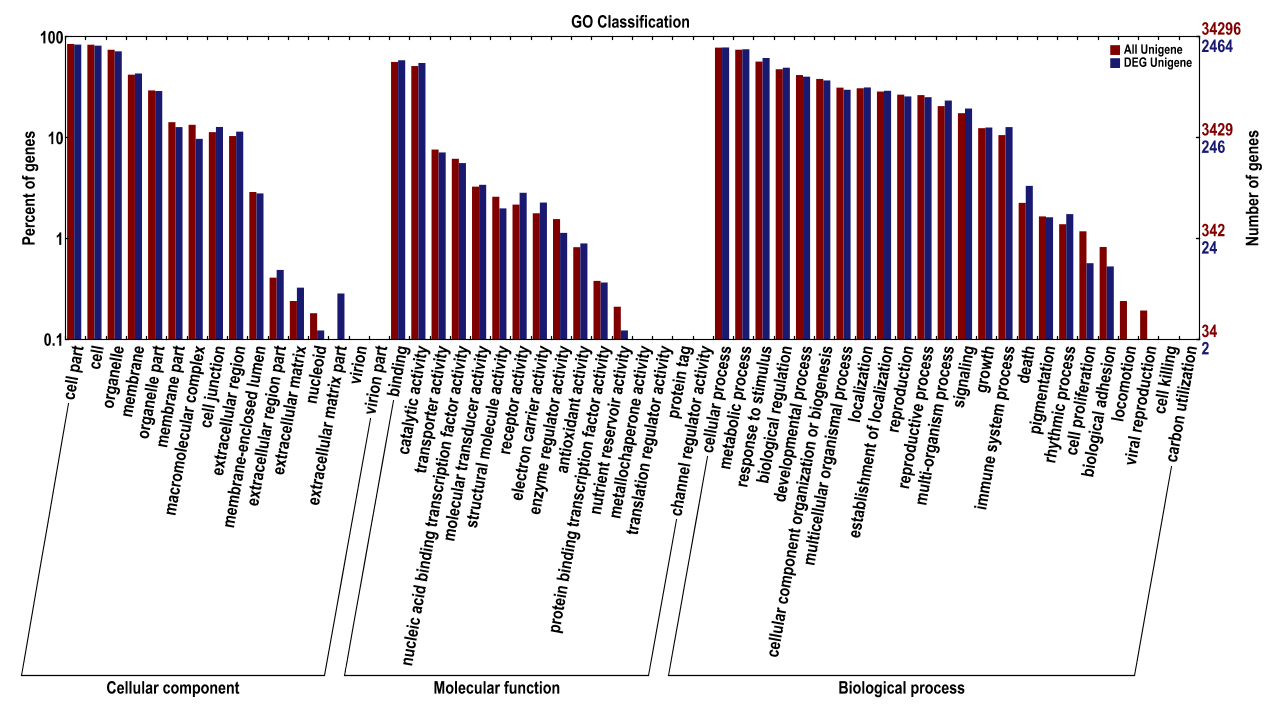

Supplement: S9 Fig — (TIF) [file pone.0135046.s009.tif]

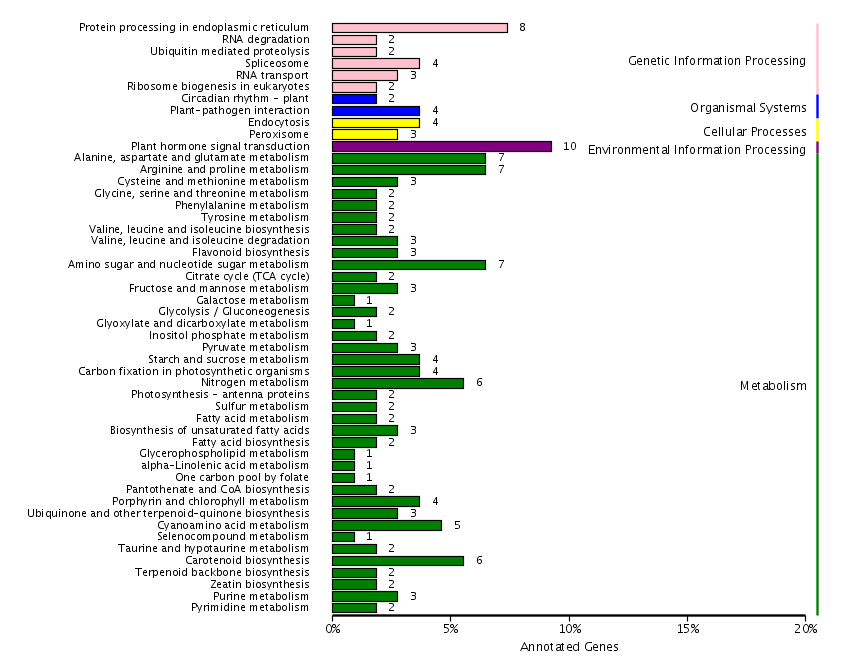

Supplement: S10 Fig — (TIF) [file pone.0135046.s010.tif]

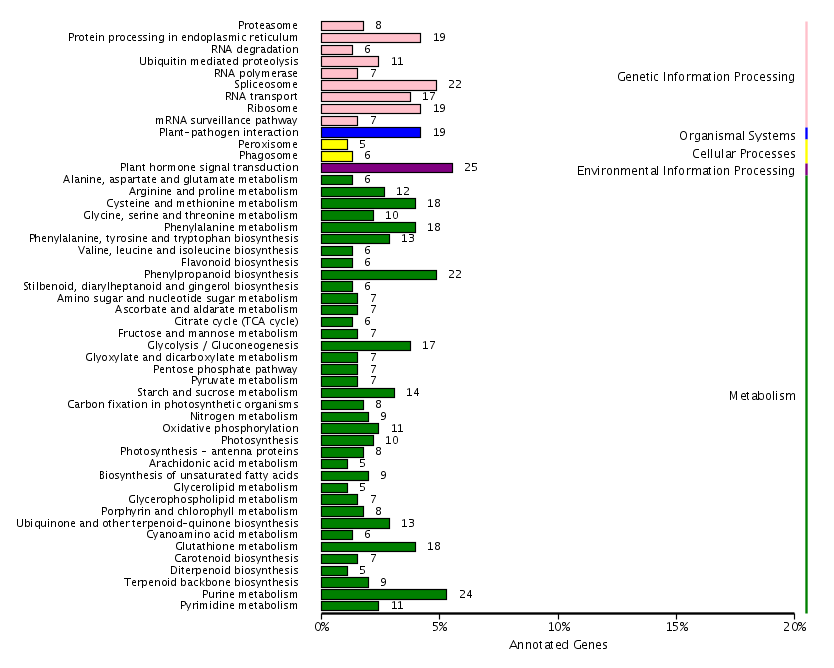

Supplement: S11 Fig — (TIF) [file pone.0135046.s011.tif]

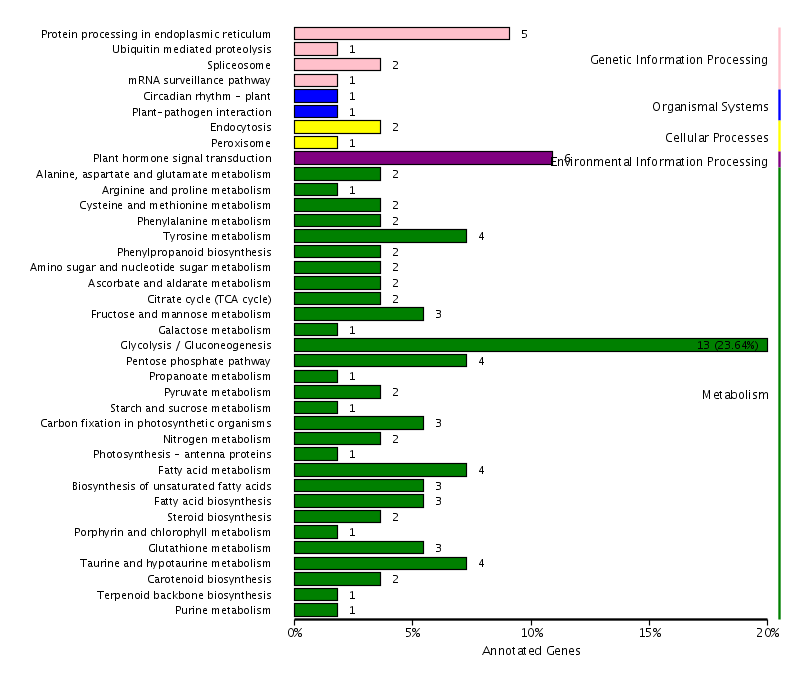

Supplement: S12 Fig — (TIF) [file pone.0135046.s012.tif]

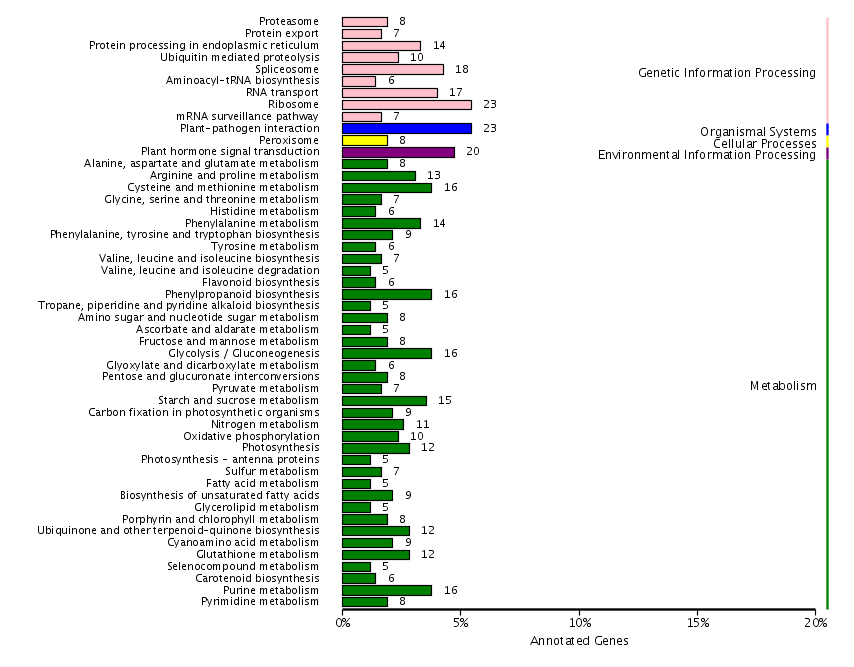

Supplement: S13 Fig — (TIF) [file pone.0135046.s013.tif]

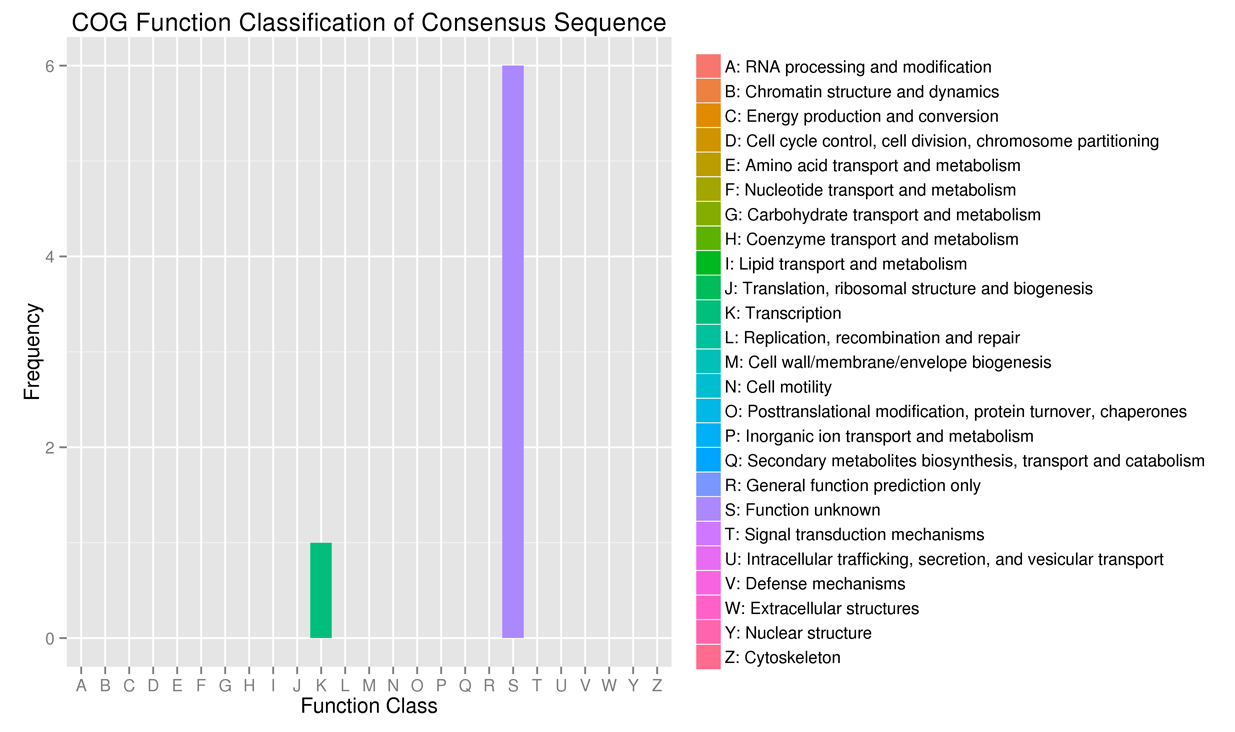

Supplement: S14 Fig — (TIF) [file pone.0135046.s014.tif]
