# Supplementary material for: Candidate Resistant Genes of Sand Pear (Pyrus pyrifolia Nakai) to Alternaria alternata Revealed by Transcriptome Sequencing
Source: PLoS One. 2015 Aug 20;10(8):e0135046. doi: 10.1371/journal.pone.0135046 (PMC4546377; doi:10.1371/journal.pone.0135046)
Supplement: S1 Table — (DOCX) [file pone.0135046.s015.docx]

S1 Table. Primers used for SSR for identify the PBS-resistant cultivars ‘Jinjing’ pear and PBS-susceptible cultivars ‘Hongfen’ pear.

| Name of primer | Primer | Lengh | Tm | Type in ‘Jinjing’ pear | Type in ‘Hongfen’ pear |
| --- | --- | --- | --- | --- | --- |
| 3436a | F：GTGGTCTTGAGGGCGTTTA | 387 | 55.2 | AB | A |
|  | R：CGTCAGCCGGTGATACTT |  | 54.9 |  |  |
| 3918 | F：TCAACTTCTTCGGCAACT | 327 | 50.3 | AB | A |
|  | R：ACGCTACCGTCATCTTTC |  | 52.6 |  |  |
| 6167b | F：GTACATGAACGCTCTGCC | 299 | 54.9 | AB | AB |
|  | R：CTCGCCTCTTATCTATCTC |  | 55.4 |  |  |
| 6484a | F：ATAAGTTGCGGCACTCTG | 392 | 52.6 | AB | A |
|  | R：CGTACCAAAAGCTAAAAT |  | 45.8 |  |  |
| 6643a | F：CTTCCAGGCAATACAAGG | 253 | 53 | A | B |
|  | R：GAGCAGCAGATTCACCAA |  | 52.6 |  |  |
| 6643d | F：CTAATCAGTCCCACAATCT | 393 | 51.3 | A | AB |
|  | R：ATCTTTGGGTACTCTTTCC |  | 50.8 |  |  |
| 12717a | F：TTTGTGATGCCTCGATAT | 316 | 48.1 | A | AB |
|  | R：ACCGTGATTCAATAGTTC |  | 50.8 |  |  |
| 12717b | F：AAACCCACTTTATCAACC | 340 | 48.1 | A | B |
|  | R：AATCTTAATAGGCGAAGG |  | 48.1 |  |  |
| 15091b | F：AGGAGGAATGAGACAATAGA | 306 | 51.3 | AB | B |
|  | R：CATCACCGTGTACCACA |  | 52.6 |  |  |
| 15091c | F：ACAACAATGGACGGAAGC | 327 | 52.6 | A | B |
|  | R：AGGAGGTGAGGGAGTGG |  | 57.2 |  |  |
| 15305a | F：AGCCCGTTAGAATGAGAT | 333 | 50.3 | A | B |
|  | R：AGCAGTATAGGCAGGGA |  | 52.6 |  |  |
