# Supplementary material for: Candidate Resistant Genes of Sand Pear (Pyrus pyrifolia Nakai) to Alternaria alternata Revealed by Transcriptome Sequencing
Source: PLoS One. 2015 Aug 20;10(8):e0135046. doi: 10.1371/journal.pone.0135046 (PMC4546377; doi:10.1371/journal.pone.0135046)
Supplement: S2 Table — (DOCX) [file pone.0135046.s016.docx]

S2 Table. Function annotation of 28 differentially expressed genes related with resistance to PBS

| Number | Gene name | Annotation |
| --- | --- | --- |
| 1 | Pbr025376 | Rosa hybrid cultivar partial brp34 gene for putative LZ-NBS-LRR resistance protein |
| 2 | Pbr025080 | PREDICTED: Fragaria vesca subsp. vesca probable disease resistance protein At5g66900-like (LOC101297940), mRNA |
| 3 | pear_newGene_1053 | Probable disease resistance protein |
| 4 | Pbr022876 | Pyrus pyrifolia clone Pp-Qh-8 nucleotide binding site leucine-rich repeat disease resistance protein gene, partial cds |
| 5 | Pbr023278 | Ricinus communis hypothetical protein, mRNA |
| 6 | Pbr012791 | Prunus dulcis clone Pdbcs-L27 putative TIR-NBS-LRR class disease resistance protein mRNA, partial cds |
| 7 | Pbr022889 | Malus x domestica disease resistance protein (ARGA-25) gene, partial sequence |
| 8 | Pbr033741 | Putative disease resistance RPP13-like protein 1 |
| 9 | pear_newGene_2235 | Pyrus x bretschneideri clone Prg1 TIR-NBS-LRR-type disease resistance-like protein mRNA, complete cds |
| 10 | Pbr022874 | Pyrus x bretschneideri clone Pb-Dshs-2 nucleotide binding site leucine-rich repeat disease resistance protein gene, partial cds |
| 11 | Pbr023136 | Malus baccata clone OLE4-3 NBS-LRR-like protein gene, partial cds |
| 12 | Pbr007974 | PREDICTED: Fragaria vesca subsp. vesca putative disease resistance RPP13-like protein 1-like (LOC101310126), mRNA |
| 13 | pear_newGene_2229 | Pyrus x bretschneideri clone Prg1 TIR-NBS-LRR-type disease resistance-like protein mRNA, complete cds |
| 14 | Pbr040608 | PREDICTED: Fragaria vesca subsp. vesca uncharacterized LOC101301594 (LOC101301594), mRNA |
| 15 | Pbr008283 | PREDICTED: Fragaria vesca subsp. vesca putative disease resistance protein RGA1-like (LOC101297569), mRNA |
| 16 | Pbr041724 | Pyrus communis partial gene for putative nucleotide binding site leucine-rich repeat disease resistance protein, clone RGA03 |
| 17 | Pbr023112 | Malus x domestica putative disease resistance gene analog NBS-LRR (FRGA-A30) gene, partial cds |
| 18 | Pbr039001 | Prunus persica putative NBS-LRR type disease resistance protein (RPM1) mRNA, complete cds |
| 19 | Pbr000678 | Malus x domestica NBS-LRR resistance gene-like protein ARGH31 gene, partial cds |
| 20 | Pbr000681 | Malus x domestica NBS-LRR resistance gene-like protein ARGH31 gene, partial cds |
| 21 | Pbr012606 | PREDICTED: Fragaria vesca subsp. vesca probable disease resistance RPP8-like protein 2-like (LOC101311937), mRNA |
| 22 | Pbr001627 | PREDICTED: Fragaria vesca subsp. vesca probable disease resistance protein At5g66900-like (LOC101308122), mRNA |
| 23 | Pbr034022 | TSA: Prunus avium Pa_00961 transcribed RNA sequence |
| 24 | pear_newGene_1262 | Pyrus x bretschneideri clone Prg1 TIR-NBS-LRR-type disease resistance-like protein mRNA, complete cds |
| 25 | Pbr012560 | Pyrus hybrid cultivar clone Ph-Xzl-2 nucleotide binding site leucine-rich repeat disease resistance protein gene, partial cds |
| 26 | Pbr038352 | TSA: Salicornia europaea Unigene2127_Se200S transcribed RNA sequence |
| 27 | Pbr035730 | Malus baccata clone OLE7-5 NBS-LRR-like protein gene, partial cds |
| 28 | Pbr001247 | Pyrus pyrifolia clone Pp-Yh-5 nucleotide binding site leucine-rich repeat disease resistance protein gene, partial cds |
