# Supplementary material for: Candidate Resistant Genes of Sand Pear (Pyrus pyrifolia Nakai) to Alternaria alternata Revealed by Transcriptome Sequencing
Source: PLoS One. 2015 Aug 20;10(8):e0135046. doi: 10.1371/journal.pone.0135046 (PMC4546377; doi:10.1371/journal.pone.0135046)
Supplement: S3 Table — (DOCX) [file pone.0135046.s017.docx]

S3 Table. Primers used for real-time quantitative RT-PCR for the verification of Illumina data.

| Gene  number | #ID | Primers | Gene  number | #ID | Primers |
| --- | --- | --- | --- | --- | --- |
| 1 | Pbr012791-F | GAGCCAACTCCACTGCTAAA | 15 | Pbr012560-F | CCCAGCGAAACTCCTGATTAT |
|  | Pbr012791-R | CTTATGTGCTTCTCCCAGATCC |  | Pbr012560-R | GCGTCTCTCCCTCTTCTATCT |
| 2 | Pbr012606-F | CCTACCACTGAGCAAAGAGAAG | 16 | Pbr022889-F | TGGATGGCTGTGACTTGTATC |
|  | Pbr012606-R | ATCATCAAGGACCACCAAACA |  | Pbr022889-R | CATGCATCACCAGTGTGAAATC |
| 3 | pear_new Gene_1262-F | CTTCCTCCATCCCACATCTTT | 17 | Pbr001247-F | GAGACACGCAACATCGAGAA |
|  | pear_new Gene_1262-R | TCGGTATTCACTGTTCGGTATG |  | Pbr001247-R | AGGTCAAGGTGCATGGAATAG |
| 4 | pear_newGene_1053-F | GGGAGAGATTGAGTTCCTTGAG | 18 | Pbr000681-F | CAGGTCTGCAAGGTGTTCTAA |
|  | pear_new Gene_1053-R | TCTGAGCTTCCGAGGGATATAA |  | Pbr000681-R | GGTGTGCAACTTCCTCTCTAC |
| 5 | Pbr022874-F | TGACCAGAGGCGATCCTATAA | 19 | Pbr034022-F | GTCCTAACGCGGTGTTTACT |
|  | Pbr022874-R | CACCATCTCGACTCAGCATAC |  | Pbr034022-R | GTTGCCGTCTTTGGAGTTATTG |
| 6 | Pbr001627-F | GTGGTGGGAGACTTGGAAATTA | 20 | Pbr038352-F | GCTGAAAGCAGCAGTGAAAG |
|  | Pbr001627-R | CCTGCTATATGGTTGGGTTCTC |  | Pbr038352-R | CACCCGTATCTTGGGTTCTAAT |
| 7 | Pbr025080-F | CTGGGAGATCACTTGTCTGTTC | 21 | Pbr040608-F | CAGGGTCGCATGTTACAGATAG |
|  | Pbr025080-R | ATTTGTGAGGCCTTGGTATGT |  | Pbr040608-R | GATGGAGAGGAAAGCAACAGAA |
| 8 | Pbr039001-F | CGGCGGATTTCAGCTTTATTTAC | 22 | Pbr008283-F | GTACATTGTTGGAAGGGAGGAG |
|  | Pbr039001-R | AGCTGGAGGACTAGAAGGATAC |  | Pbr008283-R | CAATCGCCATACCCGAAATAGA |
| 9 | Pbr022876-F | TCACCCATCACCATCCTTAATC | 23 | Pbr020071-F | GCTGGACGGTTTGGGATATTA |
|  | Pbr022876-R | ATTCTCCTTCCTCGGTGTTTC |  | Pbr020071-R | GAGGAGAGTTTGGTTCCCTTATAC |
| 10 | Pbr023278-F | CAAAGTTCGCAATCTCGCATAG | 24 | Pbr037418-F | CTCGTGTGTTGAGCCTGTAA |
|  | Pbr023278-R | ATGTGGAGGTGTGCCATTAG |  | Pbr037418-R | GATAGGTAGGCATCCGAAACAA |
| 11 | Pbr033741-F | GAATGGAAGACACGGCTAGAA | 25 | Pbr040066-F | GCGGGTATTGTAGGAGCTAAAG |
|  | Pbr033741-R | AACCGACCCAAGTCGTTAAG |  | Pbr040066-R | TCGAGGACGATGTGCTAGTTA |
| 12 | Pbr025376-F | AGGAGCCACAAACACACTAC | 26 | Pbr042781-F | TCCGTATCCGAACTCGATTAGA |
|  | Pbr025376-R | TTGGGACATGAGTTGAGGATAAG |  | Pbr042781-R | GCCAAGCAATCTCTGGTTAGA |
| 13 | Pbr041724-F | CAGTTTGGGAGTGGGCAATA | 27 | GAPDH-F | GTGCCCACTGTTGATGTTTCC |
|  | Pbr041724-R | TTGGTGAAGGAGCTGAAGATG |  | GAPDH-R | CCTTCTGACTCCTCCTTGATAGC |
| 14 | Pbr000678-F | CGAGAGTGTGAGGTGTTGTAAG |  |  |  |
|  | Pbr000678-R | GCGATGGGACAAACAGGTAT |  |  |  |
